# Comprehensive analysis suggests CRIF1 is a potential target in breast cancer associated with prognosis and immune infiltration

**DOI:** 10.1080/07853890.2025.2593151

**Published:** 2026-05-12

**Authors:** Yongping Li, Jie Liu, Ming Zhong, Weiping Yu, Hongbo Zhu, Xueting Wang, Hao Yuan

**Affiliations:** a Department of Breast and Thyroid Surgery, Shanghai Pudong Hospital, Fudan University Pudong Medical Center, Shanghai, China; b Department of Pathology, Shanghai Pudong Hospital, Fudan University Pudong Medical Center, Shanghai, China; c Department of Operating Room, Shanghai Pudong Hospital, Fudan University Pudong Medical Center, Shanghai, China

**Keywords:** CRIF1, breast cancer, immunotherapy, biomarker, prognosis

## Abstract

**Background:**

CRIF1 is a multifunctional factor that regulates cell biological processes such as the cell cycle, cell proliferation, and energy metabolism, and it is a new molecule that contributes to the poor prognosis of many malignancies. However, its involvement in breast cancer development is not fully known.

**Materials and methods:**

To investigate the relationship between CRIF1 expression, prognosis, and clinical characteristics using The Cancer Genome Atlas (TCGA-BRCA). The relationship between CRIF1 expression and the immunological microenvironment was investigated using CIBERSORT, ESTIMATE. Breast tissue and CRIF1 expression were validated by IHC. A tiny interfering plasmid was designed to transiently transfect breast cancer cell lines, and proliferation-related functional tests were carried out. The effect of sh CRIF1 on tumor formation was confirmed using a subcutaneous tumor experiment in naked mice.

**Results:**

We discovered that CRIF1 was highly elevated in breast cancer tissues and associated with a poor prognosis. CRIF1 stimulates breast cancer cell proliferation, migration, and invasion. Knockdown decreased PI3K/AKT/mTOR signaling, which boosted autophagy activity. Immune infiltration research revealed that patients with high CRIF1 expression had higher CD8^+^ T cell expression but reduced macrophage M2 expression.

**Conclusion:**

Upregulation of CRIF1 in breast cancer cells enhances malignant behavior, which may be mediated by PI3K/AKT/mTOR signaling and is linked to cellular autophagy.

## Introduction

Breast cancer (BC) is one of the most frequent malignant tumors in women, accounting for more than 24% of new cancer cases in women globally and 15% of cancer-related deaths [1]. BC treatment has advanced significantly in recent decades, although morbidity and death remain high [2,3]. Breast cancer prevalence is increasing year after year, and the prognosis for early-stage patients is reasonably excellent, however, metastatic patients have a terrible prognosis, with a 5-year survival rate of about 25% [4]. It is critical to understand the molecular mechanisms of BC proliferation and progression [5,6]. Furthermore, developing more effective coping methods and precision targeted medicines is a pressing task with significant clinical implications.

CR6 Interacting Factor 1 (CRIF1) is a new factor that regulates a variety of cellular biological processes, including the cell cycle, cell proliferation, and energy metabolism [7]. CRIF1 is best recognized for acting as a negative regulator of the cell cycle by binding directly to the Gadd45 family of proteins or CDK2 [7–9]. In addition, CRIF1 regulates multiple transcription factors, including Nur77 and STAT3, and influences cancer cell growth [10]. CRIF1 has been identified in a variety of cancer-related studies. CRIF1 accelerates the advancement of non-small-cell lung cancer by deacetylating PYCR1 *via* SIRT3 [11]. CRIF1 promotes the growth of non-small-cell lung cancer through SNF5, a chromatin remodeler in the HCT116 colon cancer cell line that increases p53 activity [12]. Overexpression of CRIF1 in hepatocellular carcinoma increases tumor development and metastasis *via* activating the ROS/NFkappaB pathway [13]. Although there has been some evidence that CRIF1 siRNA-encapsulated PLGA nanoparticles reduce tumor growth in MCF-7 human breast cancer cells [14]. However, research on CRIF1 in breast cancer as a whole is currently insufficient.

In this study, our bioinformatics analysis using The Cancer Genome Atlas (TCGA) data revealed an abnormal overexpression of CRIF1 in BC, which was confirmed by clinical samples. In addition, the link between CRIF1 and prognosis and immune infiltration was clarified. On the other hand, we used GSEA and KEGG enrichment analysis to evaluate the putative CRIF1 signaling pathways in BC progression. The variations in CRIF1 in BC stratification and drug therapy selection were determined using mutation percentage analysis and drug sensitivity analysis. Finally, we investigated the biological effects and probable mechanisms of CRIF1 in cell and animal models.

## Materials and methods

### Data and clinical sample collection

The TCGA (https://portal.gdc.cancer.gov/repository) database was used to obtain mRNA data from 113 normal and 1207 BC tissues and their corresponding clinical data, and the corresponding clinical indicators included patient age, gender, pathological stage, and TMN stage. TCGA belongs to public databases. The patients involved in the database have obtained ethical approval. Users can download relevant data for free for research and publish relevant articles. Our study is based on open datasets, so there are no ethical issues and other conflicts of interest.

The 12 BC tissues used for validation, including the corresponding adjacent non-tumor tissues, were obtained from Shanghai Pudong Hospital in March 2024, and the study protocol was approved by the Ethics Committee. All experiments were conducted in accordance with relevant regulations, and all patients provided written informed consent.

### Analysis of differential expression, prognosis, and clinicopathologic correlation of CRIF1

Significance was calculated by the Wilcox test based on the correlation analysis of CRIF1 differential expression and clinicopathologic features. Prognostic analysis was performed by GEPIA2 (http://gepia2.cancer-pku.cn/#index). The TCGA (https://portal.gdc.cancer.gov/repository) database.

### Enrichment analysis

The package ‘ClusterProfiler’ [15] was used for gene ontology (GO) enrichment and Kyoto Encyclopedia of Genes and Genomes (KEGG) pathway analysis of CRIF1-related genes. We used GSEA software [16] and analyzed the differences in signaling pathways between the high CRIF1 and low CRIF1 groups in BC. *p* < 0.05 was considered significant.

### Analysis of immune cell infiltration

Estimate [17] is a reliable method for inferring the stromal-to-immune cell ratio in tumor samples using genetic characteristics. We used Wilcoxon’s test to examine the differences between the CRIF1 and tumor microenvironment (TME) groups. CIBERSORT [18] R script was used to calculate the relative fraction of invading immune cells. We used the Spearman approach to create correlation lollipop plots for 22 different immune cell types.

### Tumor mutation profiles and drug sensitivity screening

Mutation data for tumor samples were retrieved from TCGA-GDC. Subsequently, the expression levels of CRIF1 were visualized, categorizing samples into high and low expression cohorts. Tumor mutation burden (TMB) was also calculated for these samples. The aforementioned analyses were performed utilizing the R package ‘maftools’. The dataset was further subjected to analysis using the oncoPredict [19] R software package. The average IC50 values for each chemotherapeutic agent were ascertained within both the high and low CRIF1 expression groups. To evaluate the differences in IC50 values between the two groups for each drug, the Wilcoxon Rank Sum test was applied, with a significance threshold set at *p* < 0.05.

### Cell culture and transfection

Breast cancer cell lines (MCF7, MDA-MB-231) were obtained from the Institute of Cell Science, Chinese Academy of Sciences. The cells were grown in DMEM high sugar medium (Gibico) with 10% FBS (Gibico) and 1% penicillin-streptomycin solution (Thermofisher). Cells were placed in an incubator at 37 °C with 5% CO_2_. The siRNA specifically targeting CRIF1 was prepared by Genechem Biotechnology (China). The siRNA sequence is provided in Table S1.

### qPCR assay for the expression of CRIF1

Total RNA was isolated from tissues with Trizol (Invitrogen) and quantified with the Nanodrop 2000 (ThermoFisher Scientific). The PrimeScript™ RT kit and gDNA Eraser (TaKaRa) were used for reverse transcription. The reverse transcription was performed in accordance with the manufacturer’s instructions. The SYBR^®^ Premix Ex Taq™ kit (TaKaRa) was used to produce reaction mixtures, while an ABI7500 PCR machine (Thermo Fisher Scientific) was used for qRT-PCR. GAPDH served as an internal reference. All primers are included in Table S2.

### Cell counting kit-8 analysis and wound healing test

BC cells transfected with CRIF1 siRNA were digested when 90% fusion was reached, and inoculated in 96-well culture plates at 5000 cells/well, 5 wells/group, and assayed at 0, 1, 2, 3, 4, and 5D with CCK-8 kit (Beyotime, China).

BC cells were inoculated in 6-well plates, and after transfection with CRIF1 siRNA, the cells were scraped with a 200 μL lance tip, and the cell surface was washed with serum-free medium to remove the cell debris, and the cells were observed, photographed, and recorded in the photographs under a 40x microscope, and finally the mobility of the cells in each group was calculated.

### EDU assays

Cell proliferation was measured using the EdU Assay Kit (Yeasen Biotechnology, China). BC cells were treated with EdU for 2 h before being fixed with paraformaldehyde (4%, room temperature, 30 min) per the reagent vendor’s recommendations. The cells were then permeabilized with 0.4% Triton X-100 for 10 min before being stained in the dark with an EdU staining mixture at room temperature for 30 min. The nuclei were then stained with Hoechst 33342 at room temperature for 30 min, and the pictures were examined using fluorescence microscopy.

### Transwell assays

The effects of siCRIF1 on BC cell migration were investigated. The upper chamber received a cell suspension of 1 * 10^5^ cells, whereas the lower chamber received DMEM media with 10% FBS. The cells in the upper chamber were carefully cleaned down with a cotton swab. Cells that have migrated to the filter’s lower surface are treated with 4% paraformaldehyde for 15 min before staining with 0.1% crystal violet solution for 10 min. The cells were rinsed three times with PBS, and five fields of view per plate were randomly counted and photographed using a 100x inverted microscope.

### TUNEL analysis

TUNEL assays were performed to study apoptosis in siCRIF1 BC cells or shCRIF1 tumor cells. For cells, the Tunel Apoptosis Detection Kit (Elabscience, China) was used. Cell smears were fixed with 4% paraformaldehyde for 10 min and washed three times with PBS. Incubate with proteinase K and 0.1% Triton X-100 for 5 min. Staining was done in the dark using DAPI (Beyotime).

Sections were embedded in wax blocks. For tumors, embed in wax blocks for sectioning. Proteinase K is pretreated for 15 min and then inhibited with 3% hydrogen peroxide for 5 min according to the manufacturer’s instructions, following the instructions for the *In Situ* Cell Death Assay Kit. Terminal deoxynucleotidyl transferase was applied directly to tissue sections for 1 h. Sections are then incubated with anti-digoxin peroxidase antibody for 30 min, labeled with diaminobenzidine, and then stained with hematoxylin.

### Enzyme-linked immunosorbent assay (ELISA)

CD8^+^ T cells were co-cultured with treated MCF-7 cells at a predetermined ratio. After the incubation period, the supernatant was collected by centrifugation and used as the test sample. Subsequently, a commercial human cytokine ELISA kit was used according to the manufacturer’s instructions. The procedure included incubating the samples and serially diluted standards in a pre-coated microplate, followed by thorough washing and sequential addition of a biotinylated detection antibody and enzyme conjugate (Streptavidin-HRP). After another washing step, tetramethylbenzidine (TMB) substrate was added for color development in the dark, and the reaction was terminated with a stop solution. The absorbance (OD) was immediately measured at 450 nm using a microplate reader, and the concentrations of IL-2, IFN-γ, and TNF-α in the samples were calculated based on the standard curve.

### Immunofluorescence staining

The culture supernatants of MCF-7 cells subjected to different treatments were collected and co-cultured with macrophages. Upon completion of co-cultivation, immunofluorescence staining was performed on the macrophages through the following sequential procedures: first, the macrophages were washed with PBS, then fixed with 4% paraformaldehyde, permeabilized using Triton X-100, and blocked with serum; after these steps, the macrophages were first incubated with the primary antibody against Arg-1 at 4 °C overnight, followed by incubation with the secondary antibody conjugated with Alexa Fluor 594 for 1 h at room temperature under light-shielded conditions; finally, the cell nuclei were counterstained with DAPI, and subsequently, the fluorescence intensity of Arg-1 in macrophages was observed and analyzed using a fluorescence microscope.

### Stabilizes cell line generation

GenePharma provided us with CRIF1 short hairpin RNA (shRNA) and an interference control lentivirus. Cells were distributed in 24-well plates at 1 × 10^5^ per well. An appropriate amount of viral suspension was added, and incubation was continued for 24–48 h before being screened with 2 ng/ml puromycin for 2 weeks, with the solution changed every 3 days to remove stably transfected cell lines.

### Subcutaneous tumor formation

The animal experiment was approved by the Ethics Committee of Shanghai Pudong Hospital (20240220-001), and it was finished in May 2024. Fifteen BALB/C nude mice (vitalriver, China; 4 weeks old, female, 10–12 g) were divided into 3 groups NC, CRIF1-sh1, CRIF1-sh2 (5/group). The cell density of each group was 5 × 10^6^ cells/mL, and 0.2 mL was injected subcutaneously into the back of nude mice. Continuous observation was performed, and the animals were executed after 4 weeks by chloral hydrate (4%, intraperitoneal injection), and the tumor volume and weight were measured. We confirm that all methods were followed according to the ARRIVE guidelines 2.0: updated guidelines for reporting animal research, and in accordance with the relevant guidelines and regulations.

### Statistical analysis

Correlation coefficients were calculated by Spearman/Pearson analysis. The chi-square test and Wilcoxon test were used for the analysis of differences between groups. *p* < 0.05 was considered statistically significant. All statistical analyses were performed by R (version 4.0.3).

## Results

### Correlation of CRIF1 expression, prognosis, and clinical characteristics in BC

**Figure 1.**
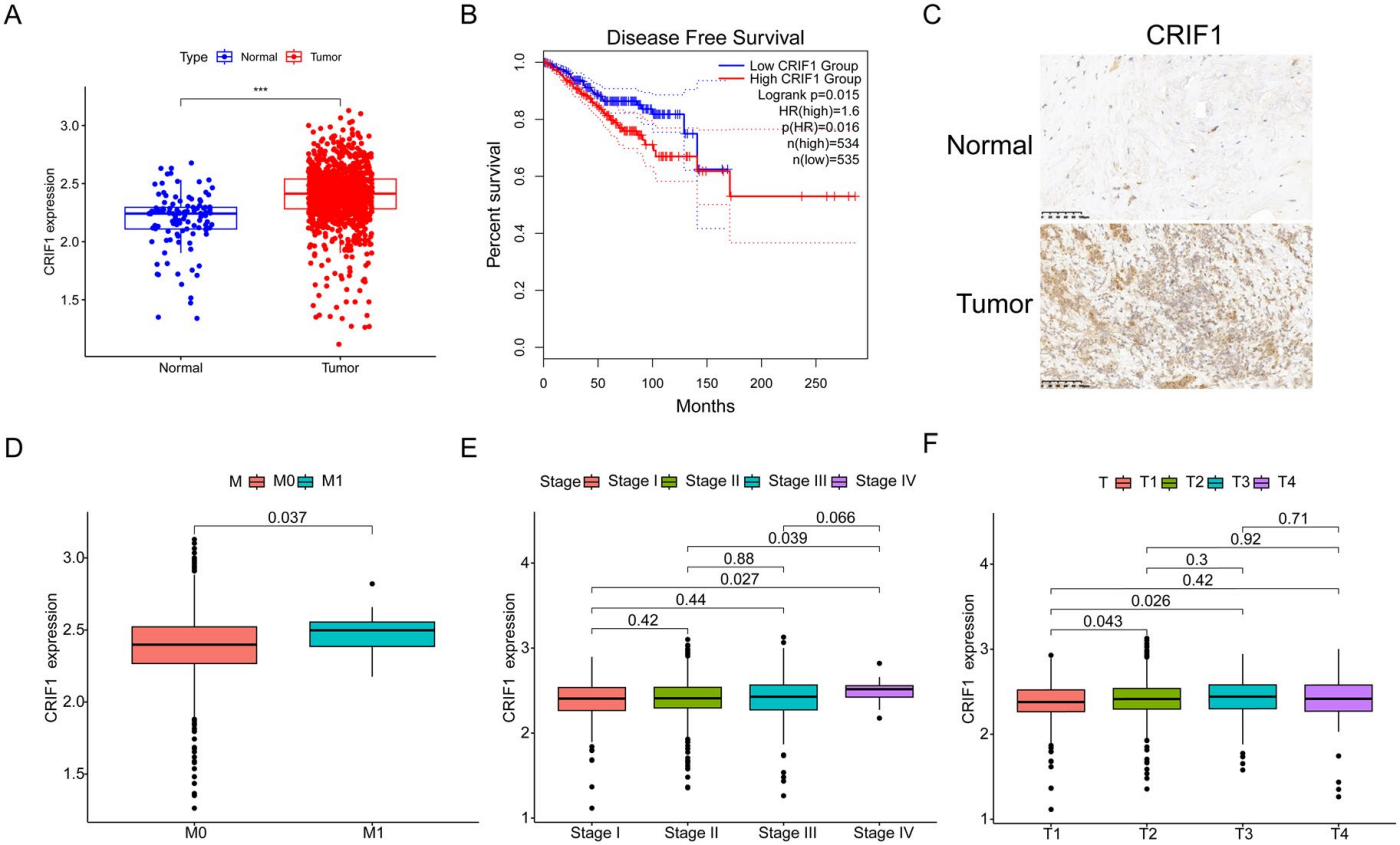
Correlation of CRIF1 expression, prognosis, and clinical characteristics in BC. (A) CRIF1 mRNA expression levels were significantly higher in breast cancer tissues compared to normal tissues, as determined by Wilcoxon rank-sum test. (B) High CRIF1 expression was associated with worse disease-free survival (DFS), as shown by Kaplan-Meier survival analysis with log-rank test. (C) Immunohistochemical staining of CRIF1 in tumor and control samples (scale: 20 µm). (D) CRIF1 expression was significantly associated with distant metastasis, as assessed by chi-square test. (E) CRIF1 expression was significantly associated with higher pathological stage (Stage IV *vs.* Stage I), as determined by chi-square test. (F) CRIF1 expression was significantly associated with larger tumor size (T3 *vs.* T1), as determined by chi-square test. ***p < 0.001.

By analyzing data from the TCGA cohort, we observed that CRIF1 expression was significantly upregulated in tumor tissues compared to normal tissues (*p* < 0.05; Figure 1A). Importantly, higher CRIF1 expression was closely linked to poorer disease-free survival (DFS) among patients (*p* < 0.05; Figure 1B). These findings were further corroborated by our analysis of data from the GEO database, specifically using the datasets GSE3744 and GSE6532, which also demonstrated consistent results with the TCGA data (Supplementary Figures 1A,B). These findings were further corroborated through immunohistochemical staining of clinically obtained breast cancer (BC) tissues, which confirmed elevated CRIF1 expression in tumor samples (Figure 1C). Additionally, our correlation analysis revealed that CRIF1 expression was associated with distant metastasis (Figure 1D), advanced pathological stage (Stage IV *vs.* Stage I; Figure 1E), and larger tumor size (T3 *vs.* T1; Figure 1F).

### Identification and functional enrichment analysis of CRIF1 signature molecules

**Figure 2.**
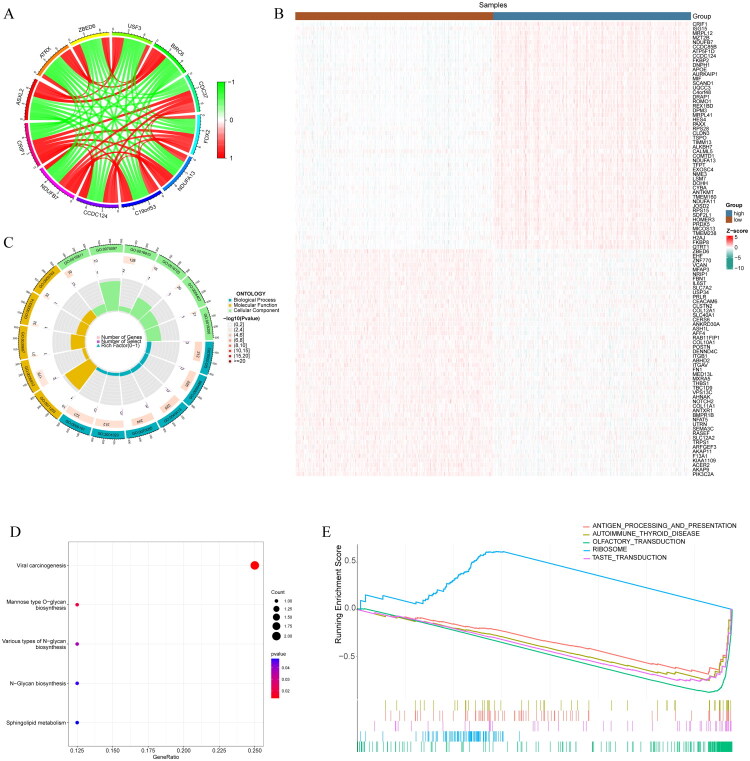
Identification and functional enrichment analysis of CRIF1 signature molecules. (A) A circle diagram showing the molecules with the highest correlation to CRIF1 in breast cancer, identified by Pearson correlation analysis. (B) A heatmap of differential genes between high and low CRIF1 groups, identified by differential expression analysis using the limma package. (C) Gene ontology (GO) enrichment analysis of CRIF1-characterized genes, performed using the ClusterProfiler package. (D) Kyoto Encyclopedia of Genes and Genomes (KEGG) enrichment analysis of CRIF1-characterized genes, performed using the ClusterProfiler package. (E) Gene set enrichment analysis (GSEA) of CRIF1-characterized genes, showing pathways differentially enriched between high and low CRIF1 groups.

In the TCGA cohort, we calculated the correlation of CRIF1 expression with other genes, and a total of 1276 (|*R*| > 0.5; *p* < 0.05) genes were identified (Table S3; Figure 2A). Meanwhile, the TCGA cohort was divided into high and low CRIF1 groups based on CRIF1 expression and differentially analyzed by limma, and a total of 1054 differentially expressed genes were obtained (Table S4; Figure 2B). The 35 intersecting genes between the two were considered to be CRIF1 signature genes (Table S5). Enrichment analysis showed that these genes were significantly enriched in biological terms such as regulation of DNA repair, protein glycosylation, and histone deacetylase activity (Figure 2C). KEGG enrichment analysis showed that CRIF1 was closely associated with O-glycan biosynthesis, N-glycan biosynthesis, and sphingolipid metabolism (Figure 2D). In addition, our KEGG-based GSEA enrichment analysis showed significant upregulation of ribosomal processes and significant inhibition of antigen processing and presentation, olfactory conductance, and taste conductance pathways (Figure 2E, Table S6).

### Relationship between CRIF1 expression and the tumor immune microenvironment

**Figure 3.**
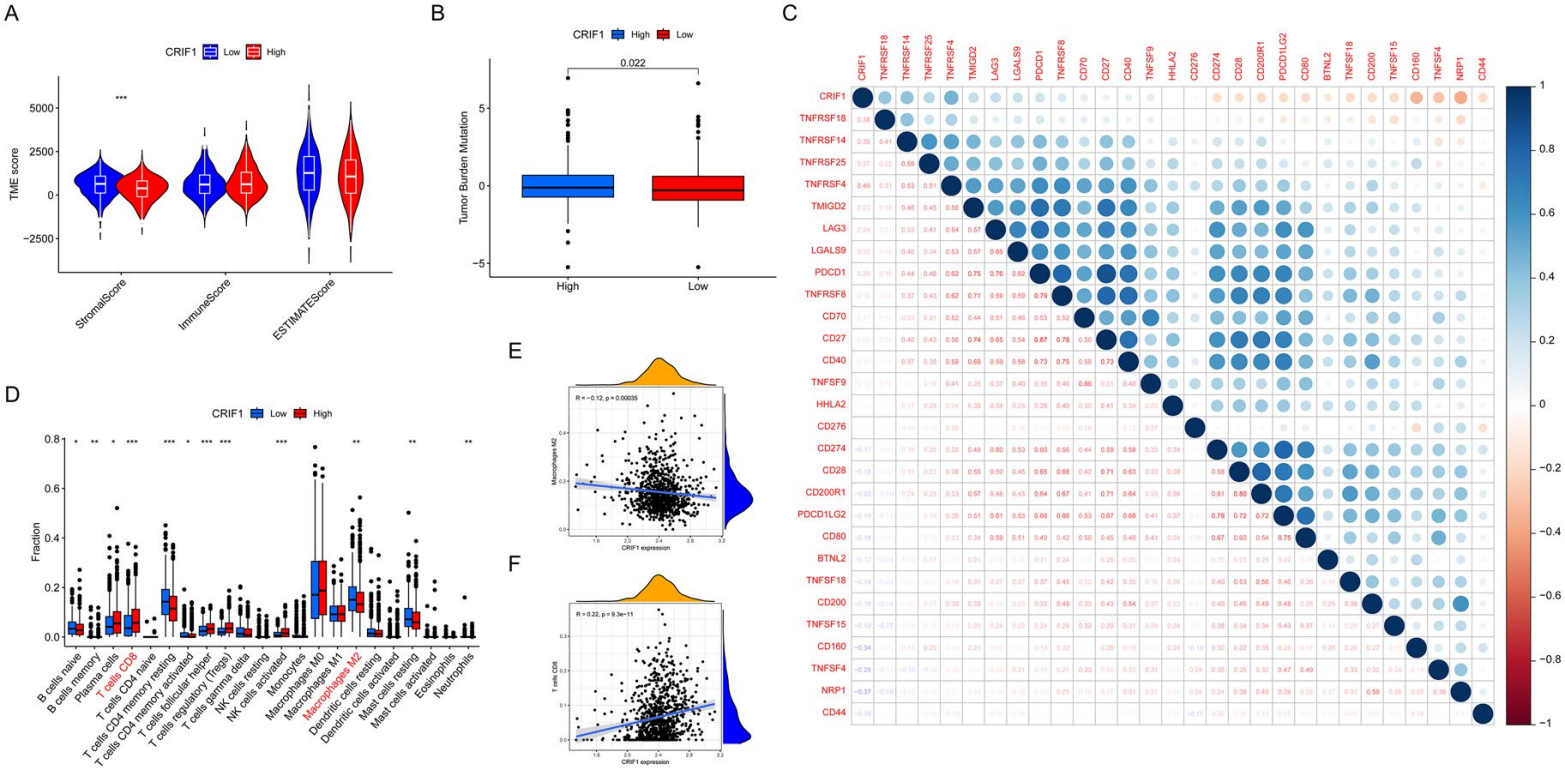
Tumor microenvironment and immune infiltration analysis. (A) CRIF1 high-expression group had lower immune and stromal scores, as determined by Wilcoxon rank-sum test. (B) Tumor mutation burden (TMB) was higher in the CRIF1 high-expression group, as determined by Wilcoxon rank-sum test. (C) CRIF1 expression was correlated with immune checkpoints, as assessed by Spearman correlation analysis. (D) CRIF1 expression was associated with the relative abundance of 22 immune cell subtypes, as determined by CIBERSORT analysis and Wilcoxon rank-sum test. (E,F) CRIF1 expression was positively correlated with CD8^+^ T cells and negatively correlated with macrophage M2, as assessed by Spearman correlation analysis. *p < 0.05, **p < 0.01 and ***p < 0.001.

To investigate the relationship between CRIF1 expression and the tumor immune microenvironment, we utilized the ESTIMATE algorithm to calculate immune and stromal scores in tumor samples. Our analysis revealed that stromal scores were significantly lower in the high-CRIF1 expression group (*p* < 0.05; Figure 3A), indicating a potential reduction in the abundance of stromal components in these tumors. Additionally, we observed that tumor mutational load (TMB) scores were significantly higher in the high-CRIF1 expression group (*p* < 0.05; Figure 3B). Given that TMB is increasingly recognized as a potential biomarker for immunotherapy response, these findings suggest that CRIF1 expression may be associated with the immunogenicity of breast cancer tumors.

We further explored the association between CRIF1 expression and key immune checkpoint molecules, identifying a general correlation (Figure 3C). This suggests that CRIF1 expression may influence the expression of immune checkpoints, which are critical for immune evasion in cancer.

To gain deeper insights into the immune cell composition associated with CRIF1 expression, we analyzed the relative abundance of 22 immune cell subtypes. Our results showed that tumors with high CRIF1 expression had a significantly higher proportion of CD8^+^ T cells (Figure 3D), which are crucial for antitumor immunity. Conversely, these tumors exhibited a lower proportion of M2 macrophages, which are known for their immunosuppressive functions (Figure 3D). Correlation analysis further confirmed these findings, demonstrating a positive correlation between CRIF1 expression and CD8^+^ T cell infiltration, and a negative correlation with M2 macrophage infiltration (Figures 3E,F). Consistent with these results, our analysis of the GSE3744 dataset from the GEO database also revealed a significant positive correlation between CRIF1 expression and CD8^+^ T cell infiltration, as well as a significant negative correlation with M2 macrophage infiltration (Supplementary Figure 2).

### Tumor mutation, drug sensitivity, and regulatory network analysis of CRIF1

**Figure 4.**
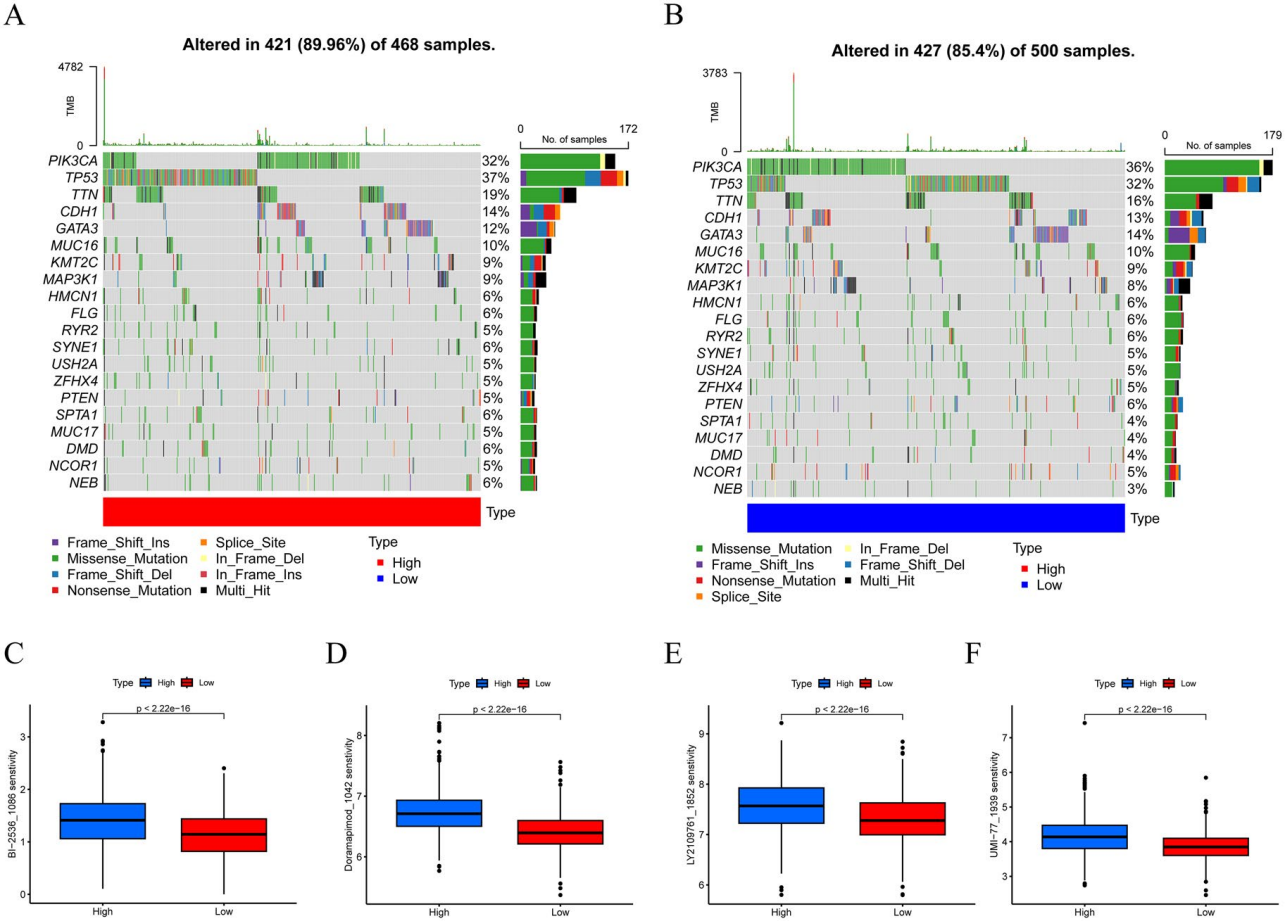
Mutation analysis and drug sensitivity analysis of CRIF1. (A,B) Mutation frequencies of the 20 most significantly mutated genes were compared between high and low CRIF1 groups using Fisher’s exact test. (C–F) Drug sensitivity analysis showed that BI-2536_1086, Doramapimod_1042, LY2109761_1852, and UMI-77_1939 were more sensitive in the high CRIF1 group, as determined by Wilcoxon rank-sum test.

We sought to analyze the association between CRIF1 and the mutations. Significantly mutated genes are shown separately according to the median value of CRIF1. Notably, among the 20 genes with the highest mutation frequency in the high CRIF1 group, the mutation frequency of TP53 was increased, whereas the mutation frequency of PIK3CA was decreased (Figures 4A,B). To screen drugs suitable for CRIF1 stratification, we evaluated the role of CRIF1 in chemotherapy or targeted therapy. BI-2536_1086, Doramapimod_1042, LY2109761_1852, and UMI-77_1939 were more sensitive in the high CRIF1 group (Figures 4C–F), which suggests that high CRIF1 expressing patients may benefit from this.

Additionally, we constructed a CRIF1-related regulatory network based on mRNA-miRNA and lncRNA-miRNA interactions. This network includes 10 miRNAs and 3 lncRNAs that interact with CRIF1, providing preliminary insights into the potential regulatory mechanisms involving CRIF1 in breast cancer (Supplementary Figure 3).

### CRIF1 knockdown inhibits breast cancer cell proliferation and modulates immune microenvironment

**Figure 5.**
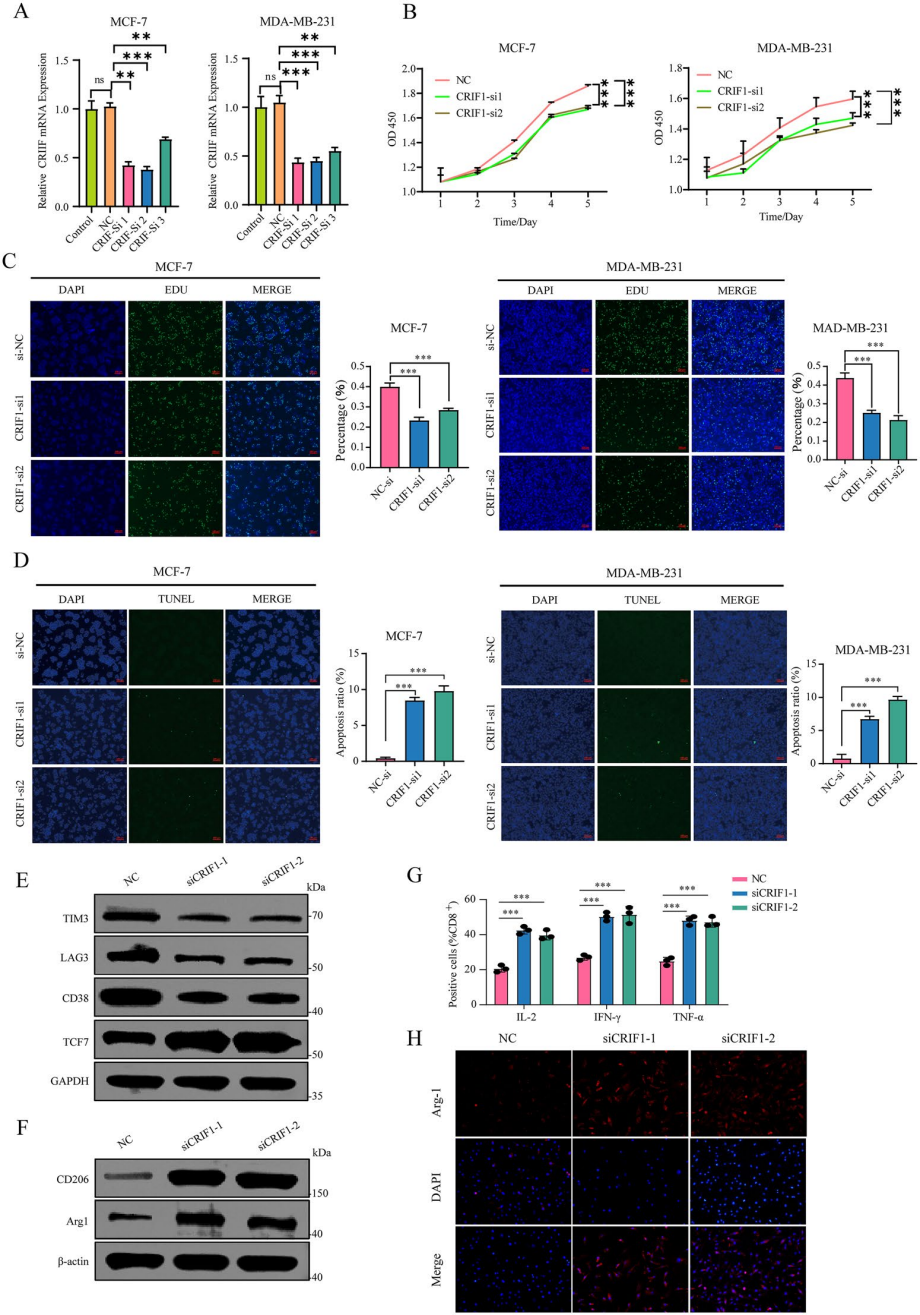
Validation of CRIF1 siRNA interference effect and proliferation results. (A) CRIF1 expression was significantly reduced in siRNA-transfected cells compared to control cells, as determined by paired *t*-test. (B) Proliferation rates were significantly slower in CRIF1 siRNA-transfected cells, as determined by one-way ANOVA with Tukey’s post-hoc test. (C) The proportion of EDU-positive cells was significantly lower in CRIF1 siRNA-transfected cells, as determined by one-way ANOVA with Tukey’s post-hoc test. (D) The number of apoptotic cells was significantly higher in CRIF1 siRNA-transfected cells, as determined by one-way ANOVA with Tukey’s post-hoc test. (E) Western Blot analysis revealed significant alterations in the expression of proteins associated with CD8^+^ T cell activation (TIM3, LAG3, CD38) in CRIF1 siRNA-transfected cells, as determined by densitometry analysis. (F) Western Blot analysis showed significant changes in the expression of proteins associated with M2 macrophage polarization (CD206, Arg-1). (G) ELISA experiments measured elevated levels of IL-2, IFN-γ, and TNF-α. (H) Arg-1-DAPI fluorescence imaging showed increased Arg-1 expression in macrophages, as determined by fluorescence intensity analysis. **p < 0.01 and ***p < 0.001.

We transiently transfected siRNA against CRIF1 (Si1, Si2, Si3) or siCtrl (NC) into MCF-7 and MDA-MB-231 cells. RT-qPCR confirmed reduced CRIF1 expression in siRNA-transfected cells, with Si1 and Si2 showing superior interference (Figure 5A). The CCK-8 assay revealed significantly slowed proliferation in these cells post-CRIF1 downregulation (Figure 5B). EDU incorporation analysis indicated fewer proliferating cells in the CRIF1 interference group (Figure 5C), while TUNEL staining showed increased apoptosis (Figure 5D). Western Blot analysis demonstrated that CRIF1 knockdown altered the expression of proteins linked to CD8^+^ T cell activation (TIM3, LAG3, CD38) and M2 macrophage polarization (CD206, Arg-1), highlighting CRIF1’s role in immune modulation (Figures 5E,F). ELISA experiments measured elevated levels of IL-2, IFN-γ, and TNF-α, further supporting CRIF1’s immune regulatory function (Figure 5G). Arg-1-DAPI fluorescence imaging showed increased Arg-1 expression in CRIF1 knockdown cells (Figure 5H). Collectively, these results demonstrate that CRIF1 promotes breast cancer cell proliferation and survival while modulating the immune microenvironment, underscoring its potential as a therapeutic target.

**Figure 6.**
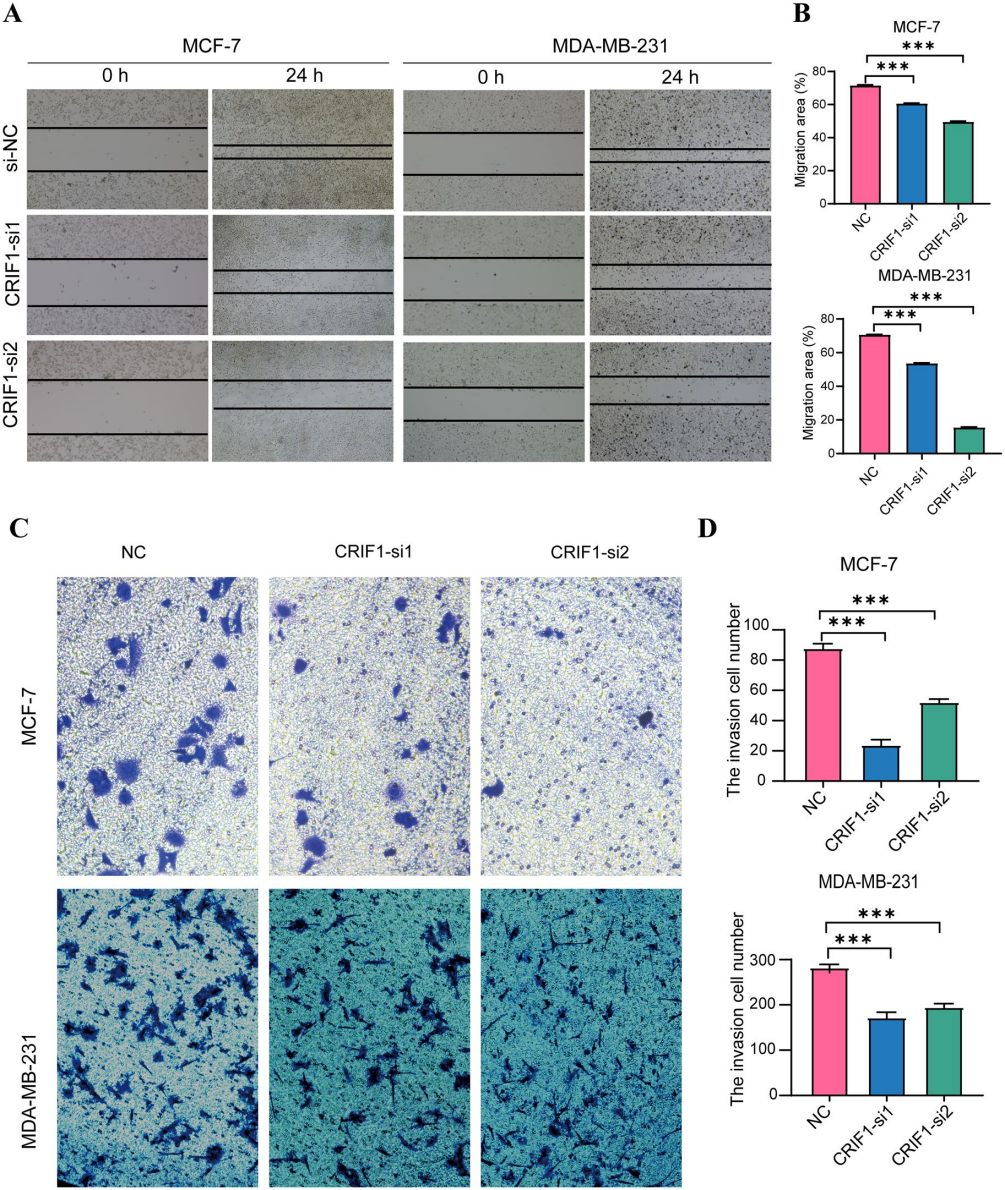
CRIF1 with the ability of BC cell migration and invasion. (A,B) Wound healing rates were significantly slower in CRIF1 siRNA-transfected cells, as determined by one-way ANOVA with Tukey’s post-hoc test. (C,D) The number of migrated cells was significantly lower in CRIF1 siRNA-transfected cells, as determined by one-way ANOVA with Tukey’s post-hoc test. ***p < 0.001.

Further, the results of scratch healing and invasion assays showed that the migratory and invasive abilities of BC cells were significantly reduced after interference with siRNA (Figures 6A–D).

### Knockdown of CRIF1 affects the expression of PI3K-AKT pathway-related genes

**Figure 7.**
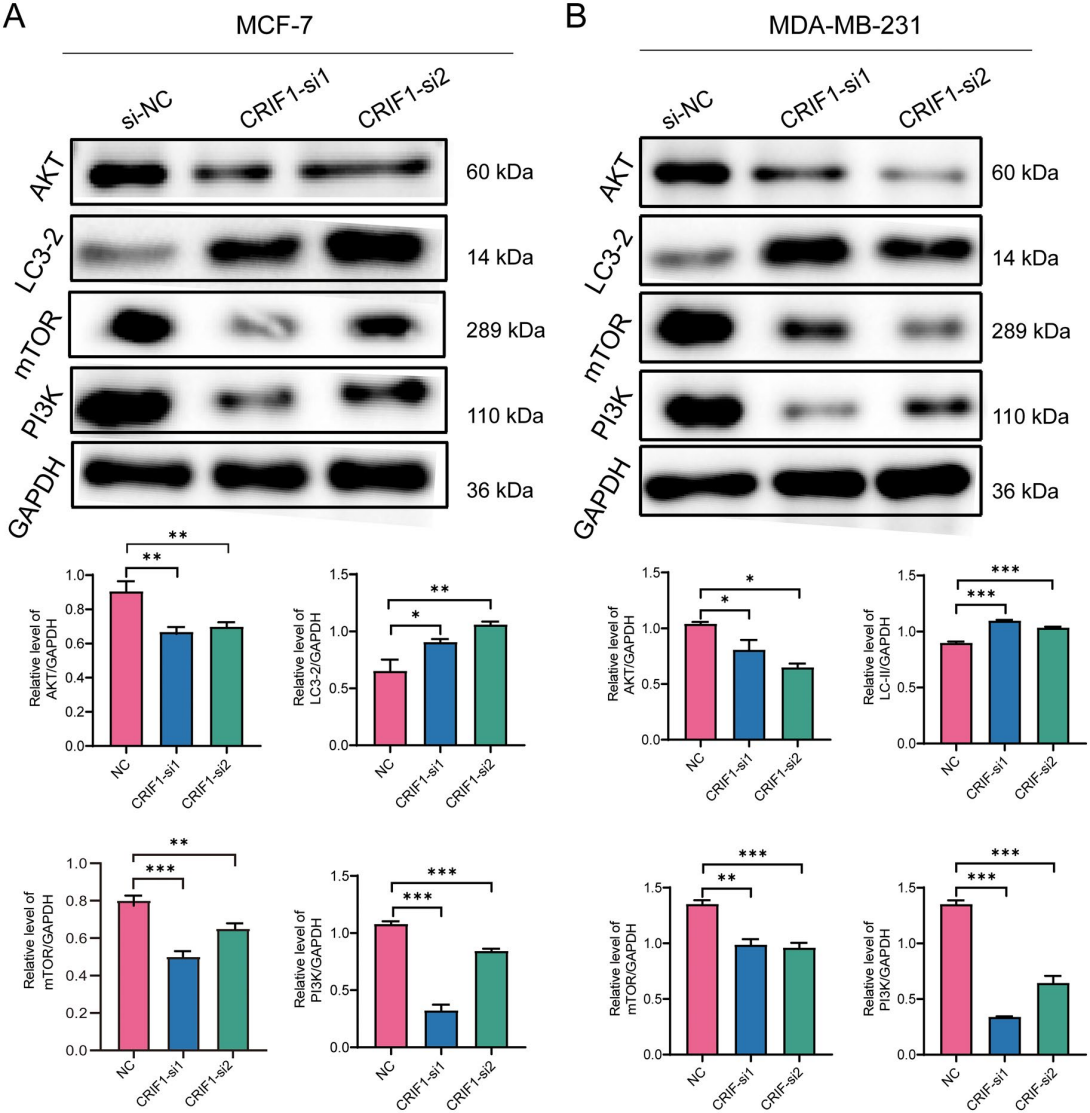
CRIF1 may mediate BC cell apoptosis through the PI3K/AKT/mTOR pathway. (A) WB detection of key PI3K-AKT signaling molecule expression in MCF-7 cells showed significant alterations following CRIF1 knockdown (one-way ANOVA with Tukey’s post-hoc test). (B) WB detection of key PI3K-AKT signaling molecule expression in MDA-MB-231 cells showed significant alterations following CRIF1 knockdown (one-way ANOVA with Tukey’s post-hoc test). *p < 0.05, **p < 0.01 and ***p < 0.001.

Previous reports have shown that phosphorylation levels of Akt and CREB are reduced in CRIF1-silenced cells (Figure 7). The Akt signaling pathway has been implicated in the changes in endothelial cell migration induced by CRIF1 downregulation [20]. And Akt signaling plays a key role in regulating tumor growth and metastasis. By WB experiments, we found that the PI3K/AKT/mTOR signaling pathway was significantly inhibited after CRIF1 silencing. In addition, autophagy level was significantly increased. This suggests that the activity of PI3K/AKT/mTOR signaling pathway was reduced, mTORC1 was inhibited, autophagy process was activated, and the conversion of LC3-I to LC3-II was increased, which promotes the formation of autophagosomes [21,22].

### In vivo *loss-of-function experiments with CRIF1*

**Figure 8.**
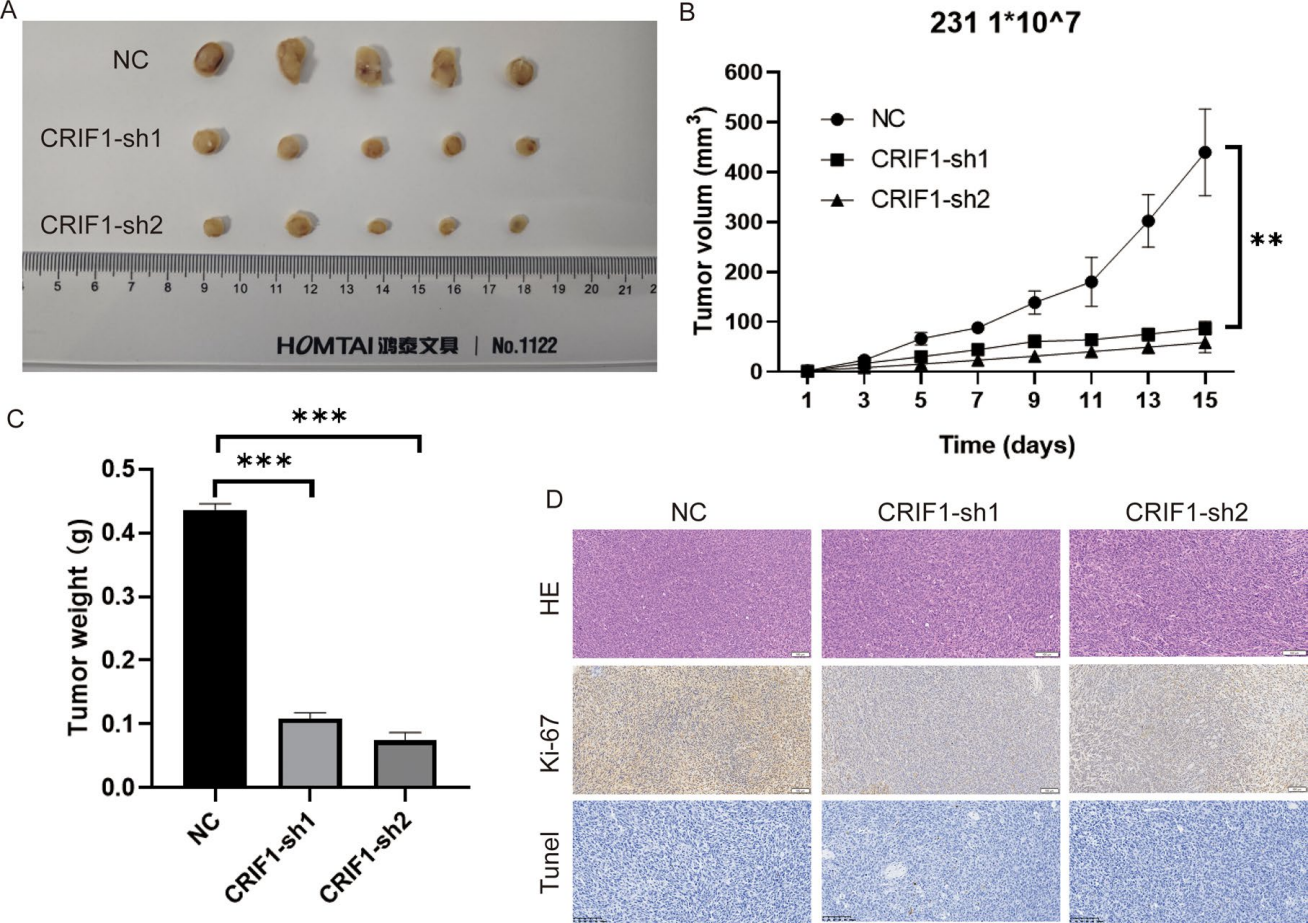
*In vivo* loss-of-function experiments with CRIF1. (A–C) Tumor size, volume, and weight were significantly smaller in CRIF1 knockdown mice, as determined by one-way ANOVA with Tukey’s post-hoc test. (D) KI-67 staining intensity was lower and TUNEL staining intensity was higher in CRIF1 knockdown tumors, as determined by one-way ANOVA with Tukey’s post-hoc test. **p < 0.01 and ***p < 0.001.

The effect of CRIF1 on BC cell growth was examined *in vivo* using a nude mouse subcutaneous tumor model. Nude mice injected with shRNA CRIB1 cells formed tumors of smaller size, volume, and weight compared with negative controls (Figures 8A–C). Correspondingly, inhibition of CRIF resulted in a significant decrease in Ki-67 expression and a significant increase in the number of TUNEL-staining positive cells (Figure 8D).

## Discussion

In our study, we first analyzed breast cancer samples from the TCGA database and found that CRIF1 expression is significantly higher in tumor tissues compared to normal tissues (Figure 1A). High CRIF1 expression is also significantly associated with poorer disease-free survival (DFS) (Figure 1B). Additionally, CRIF1 expression levels are significantly correlated with distant metastasis (Figure 1D), advanced pathological stage (Figure 1E), and larger tumor size (Figure 1F). These results indicate that overexpression of CRIF1 is closely related to the progression of breast cancer and poor prognosis. This finding is consistent with previous studies, which have shown that CRIF1, as a key regulator of the cell cycle and proliferation, is often associated with poor prognosis in various cancers [7,23].

In terms of the tumor immune microenvironment, our analysis showed that patients with high CRIF1 expression had a higher proportion of CD8^+^ T cells and a lower proportion of M2 macrophages in their tumors (Figures 3D–F). This suggests that CRIF1 may influence the progression of breast cancer by regulating the tumor immune microenvironment. Specifically, high CRIF1 expression may promote the formation of an immunosuppressive microenvironment, thereby inhibiting antitumor immune responses. This finding is consistent with recent studies showing that mitochondrial dysfunction is associated with immune suppression and immune evasion [24]. In addition, the high expression of CRIF1 in tumor cells may inhibit antitumor immune responses by affecting metabolic reprogramming [25]. These results indicate that CRIF1 not only plays a role in the biological behavior of tumor cells but also promotes tumor progression by regulating the immune microenvironment.

We further investigated the function of CRIF1 in breast cancer cells through *in vitro* experiments. Knockdown of CRIF1 significantly inhibits the proliferation (Figure 5B), migration (Figure 6A), and invasion capabilities (Figure 6B) of MCF-7 and MDA-MB-231 cells. These results suggest that CRIF1 plays a key role in the malignant behavior of breast cancer cells. The oncogenic effects of CRIF1 may be achieved by regulating cell cycle and proliferation-related pathways, which is similar to the mechanisms of CRIF1 in other cancers [7,13].

We further explored the molecular mechanisms of CRIF1 in breast cancer cells. Western blot experiments showed that knockdown of CRIF1 significantly inhibits the activity of the PI3K/AKT/mTOR signaling pathway and increases autophagy levels (Figure 7). These results suggest that CRIF1 may regulate the proliferation and survival of breast cancer cells through the PI3K/AKT/mTOR signaling pathway, with autophagy activation being one of its downstream effects. The PI3K/AKT/mTOR signaling pathway has been widely studied in various cancers, and its activation is usually associated with cell proliferation, survival, and drug resistance [21,22]. Our study results further support the hypothesis that CRIF1 exerts its oncogenic effects in breast cancer through this pathway.

Finally, we validated the effect of CRIF1 on tumor growth in a nude mouse model. Tumors with CRIF1 knockdown had significantly reduced volume and weight (Figures 8A–C), and lower Ki-67 expression and higher TUNEL-positive cell counts (Figure 8D). These results further confirm the oncogenic role of CRIF1 in breast cancer and suggest that CRIF1 may be a potential therapeutic target for breast cancer. These *in vivo* experimental results are consistent with the *in vitro* experimental results, further supporting the key role of CRIF1 in the progression of breast cancer.

Despite our study providing evidence for the important role of CRIF1 in breast cancer, there are still some limitations. First, our analysis is mainly based on data from the TCGA database. Although these data are widely representative, they may not fully cover the characteristics of all breast cancer patients. Second, our experiments mainly focused on two cell lines, MCF-7 and MDA-MB-231, which represent hormone receptor-positive and triple-negative breast cancer subtypes, respectively. However, other subtypes (such as HER2-positive breast cancer) were not included in our study. In addition, our sample size is relatively small, which may affect the power of statistical analysis. Future research needs to validate these findings in more independent clinical samples and databases to ensure their reliability and generalizability. Furthermore, additional functional experiments, including investigations into the mechanisms of CRIF1 in other breast cancer subtypes, will help to gain a more comprehensive understanding of the biological functions of CRIF1 in breast cancer.

In summary, our study demonstrates that overexpression of CRIF1 in breast cancer is closely related to tumor progression, changes in the immune microenvironment, and poor prognosis. CRIF1 regulates the proliferation and survival of breast cancer cells through the PI3K/AKT/mTOR signaling pathway and may promote tumor progression by affecting the immune microenvironment. These findings provide a theoretical basis for developing new therapeutic strategies targeting CRIF1. Future research will focus on further validating the role of CRIF1 in different breast cancer subtypes and exploring its potential as a therapeutic target. In addition, we plan to validate these findings in more clinical samples and independent databases to ensure their reliability and generalizability.

## Supplementary Material

Supplementary Figure 3.tif

Supplementary Tables.xlsx

Supplementary Figure 2.tif

Supplementary Information WB raw data.docx

ARRIVE guidelines.pdf

Supplementary Figure 1.tif

## Data Availability

The data that support the findings of this study are available from the corresponding author, [HY], upon reasonable request.
