# Supplementary figures and images for: Comprehensive analysis suggests CRIF1 is a potential target in breast cancer associated with prognosis and immune infiltration
Source: Ann Med. 2026 May 12;58(1):2593151. doi: 10.1080/07853890.2025.2593151 (PMC13169454; doi:10.1080/07853890.2025.2593151)

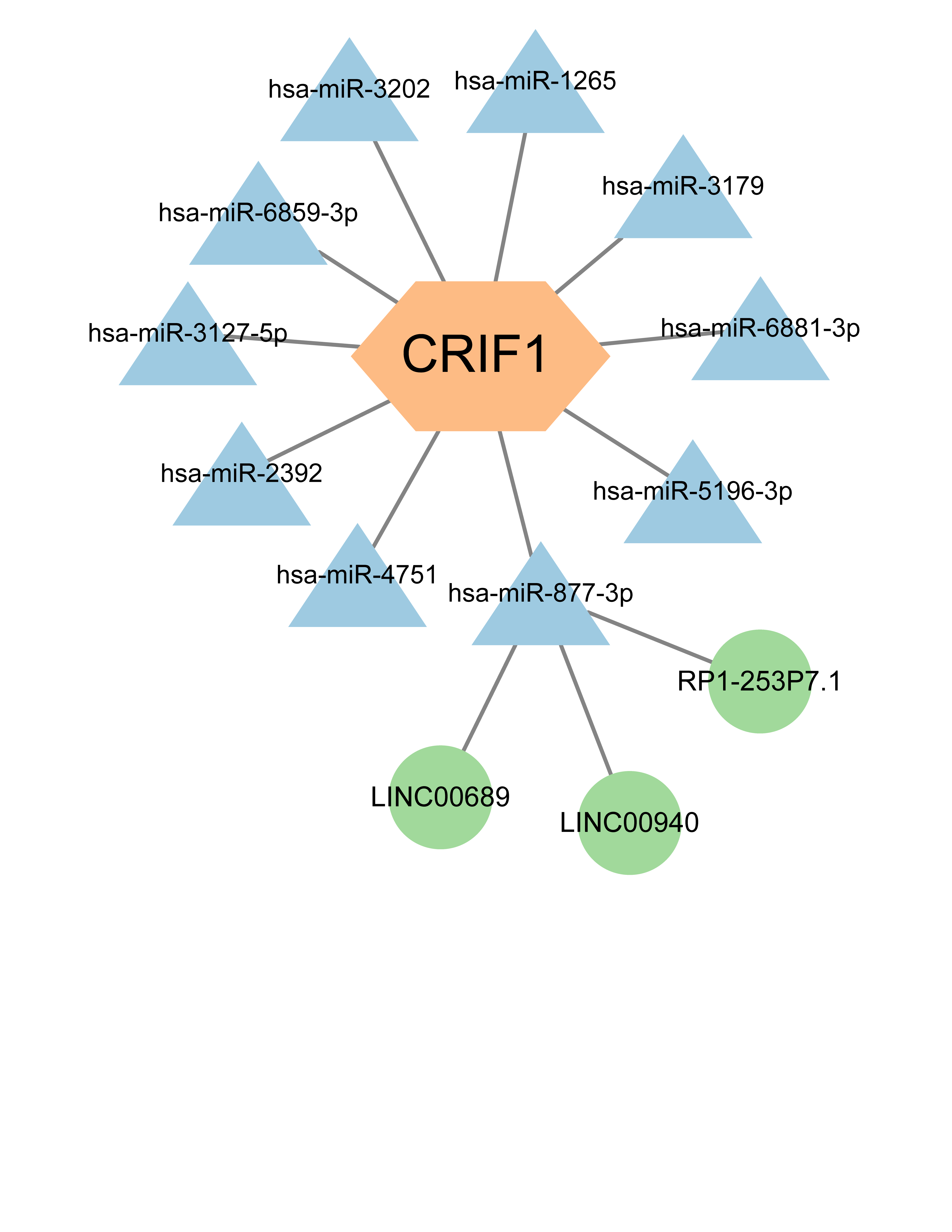

Supplement: Supplementary Figure 3.tif [file IANN_A_2593151_SM3485.tif]

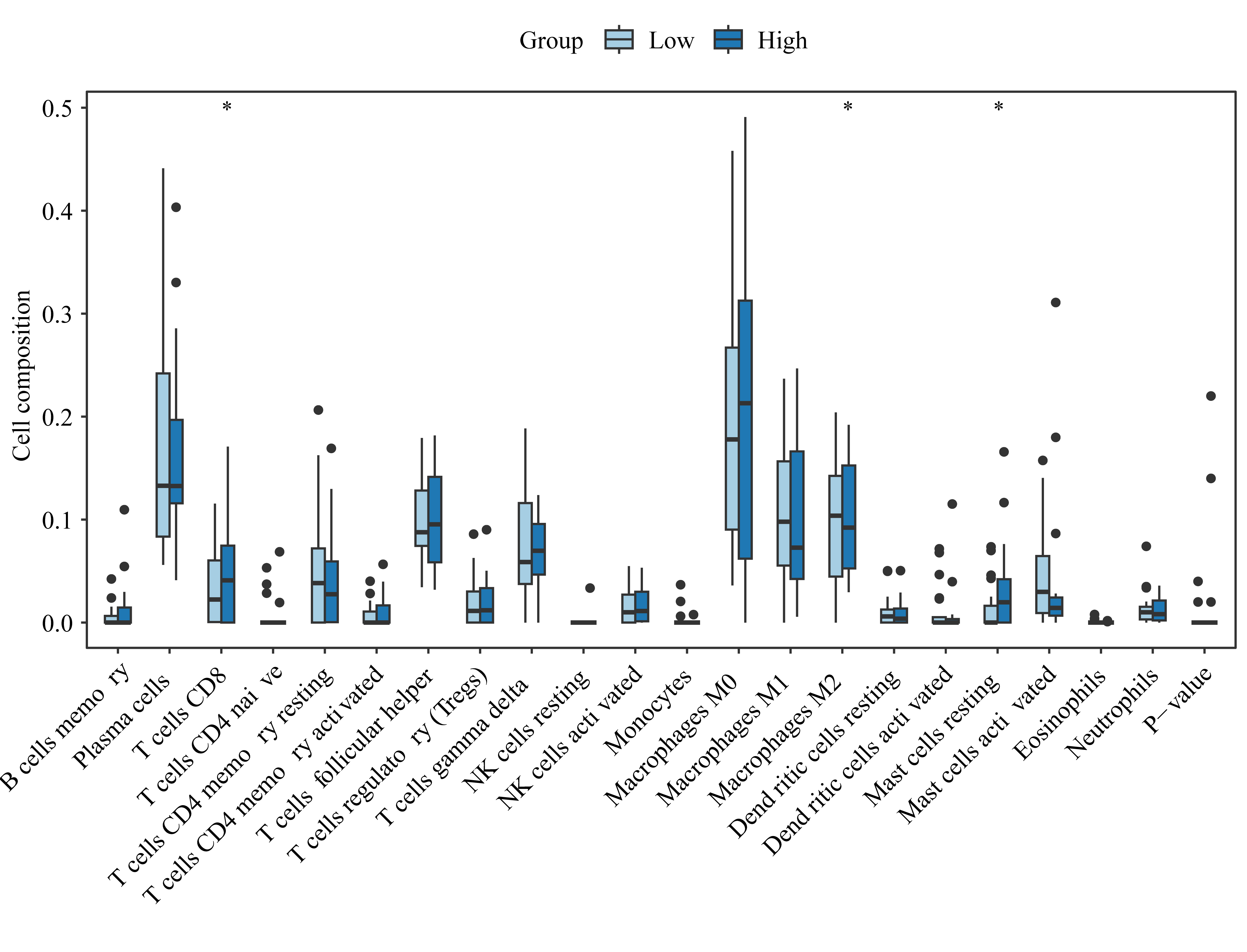

Supplement: Supplementary Figure 2.tif [file IANN_A_2593151_SM3483.tif]

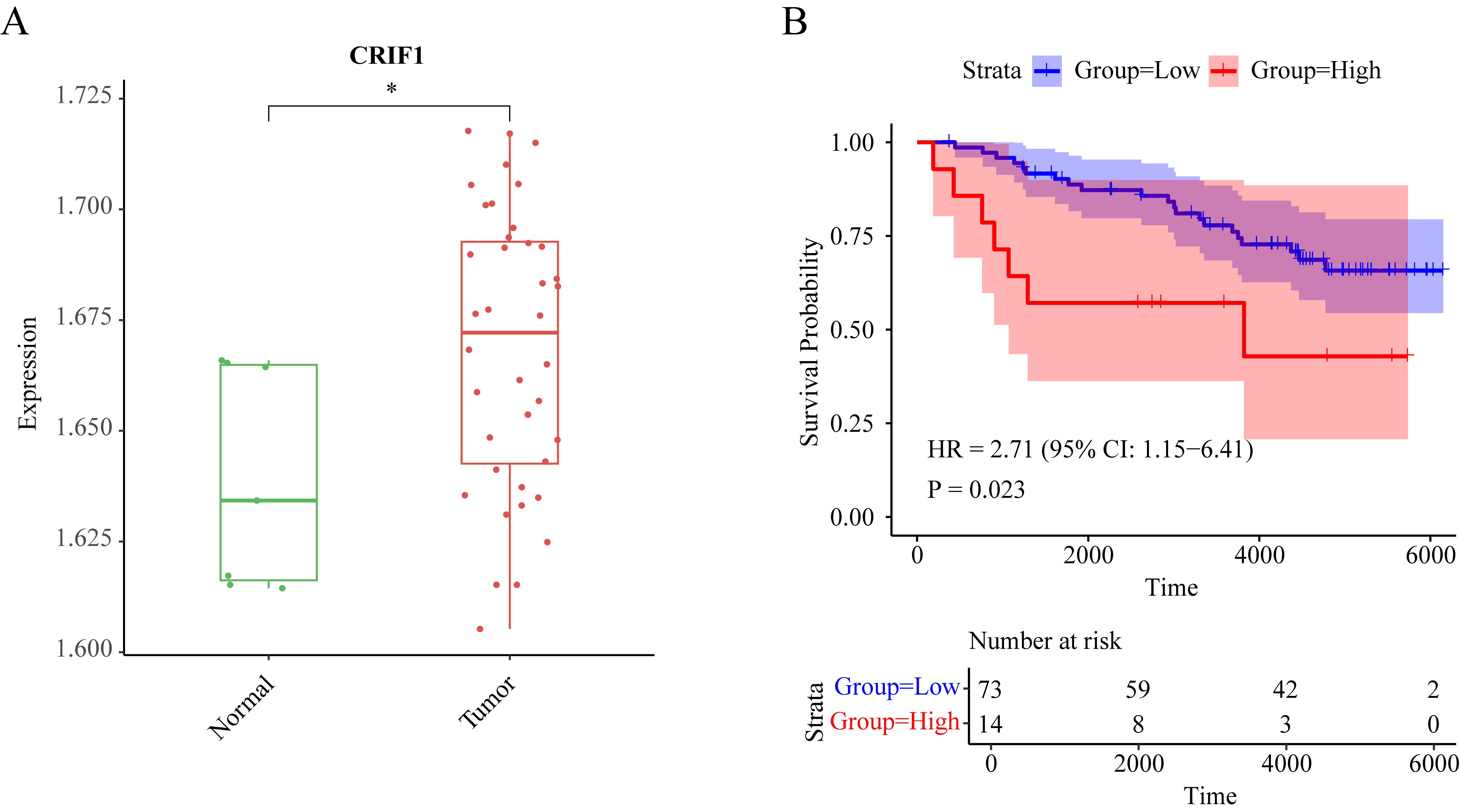

Supplement: Supplementary Figure 1.tif [file IANN_A_2593151_SM3480.tif]
