# Supplementary material for: Comprehensive analysis suggests CRIF1 is a potential target in breast cancer associated with prognosis and immune infiltration
Source: Ann Med. 2026 May 12;58(1):2593151. doi: 10.1080/07853890.2025.2593151 (PMC13169454; doi:10.1080/07853890.2025.2593151)
Supplement: Supplementary Information WB raw data.docx [file IANN_A_2593151_SM3482.docx]

**Extended Data: The uncropped, original Western blot images corresponding to the main figures.**


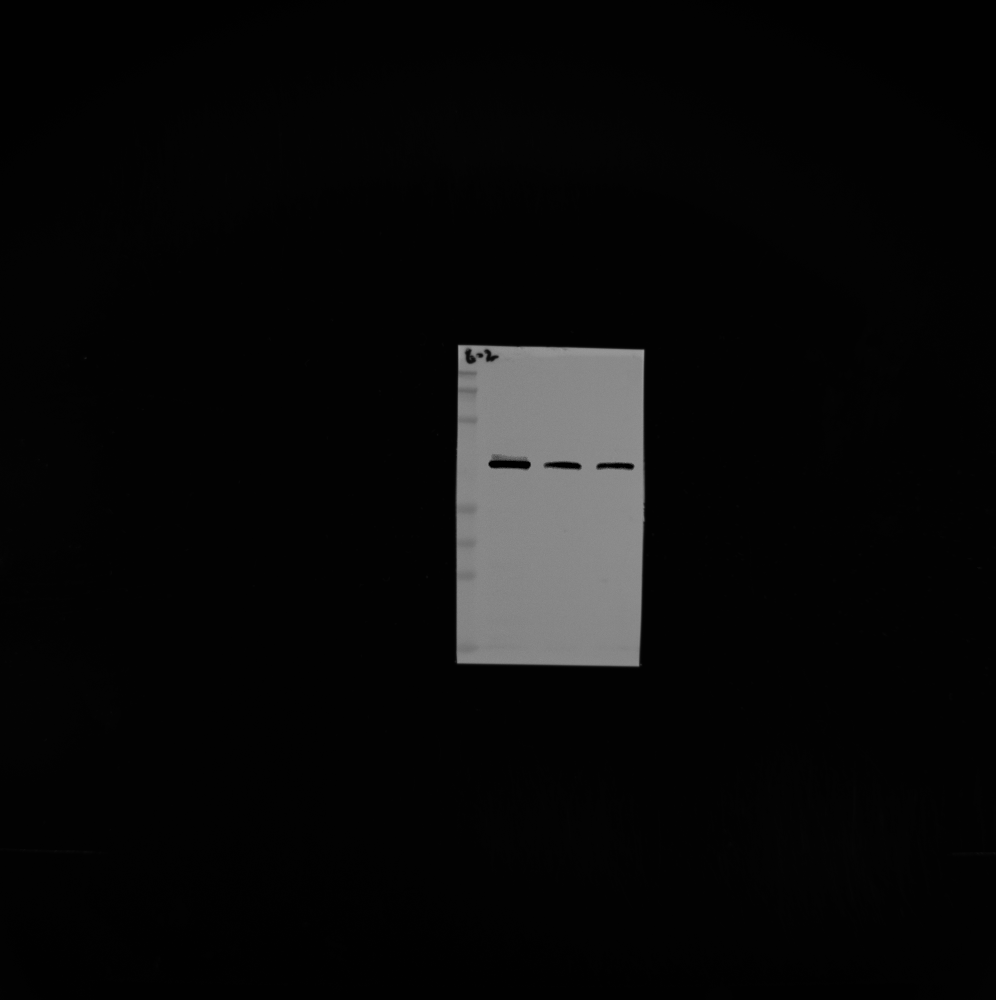


TIM-3 70kDa

**Extended Data Fig.1** The original blotting of TIM-3 in Fig.5E.


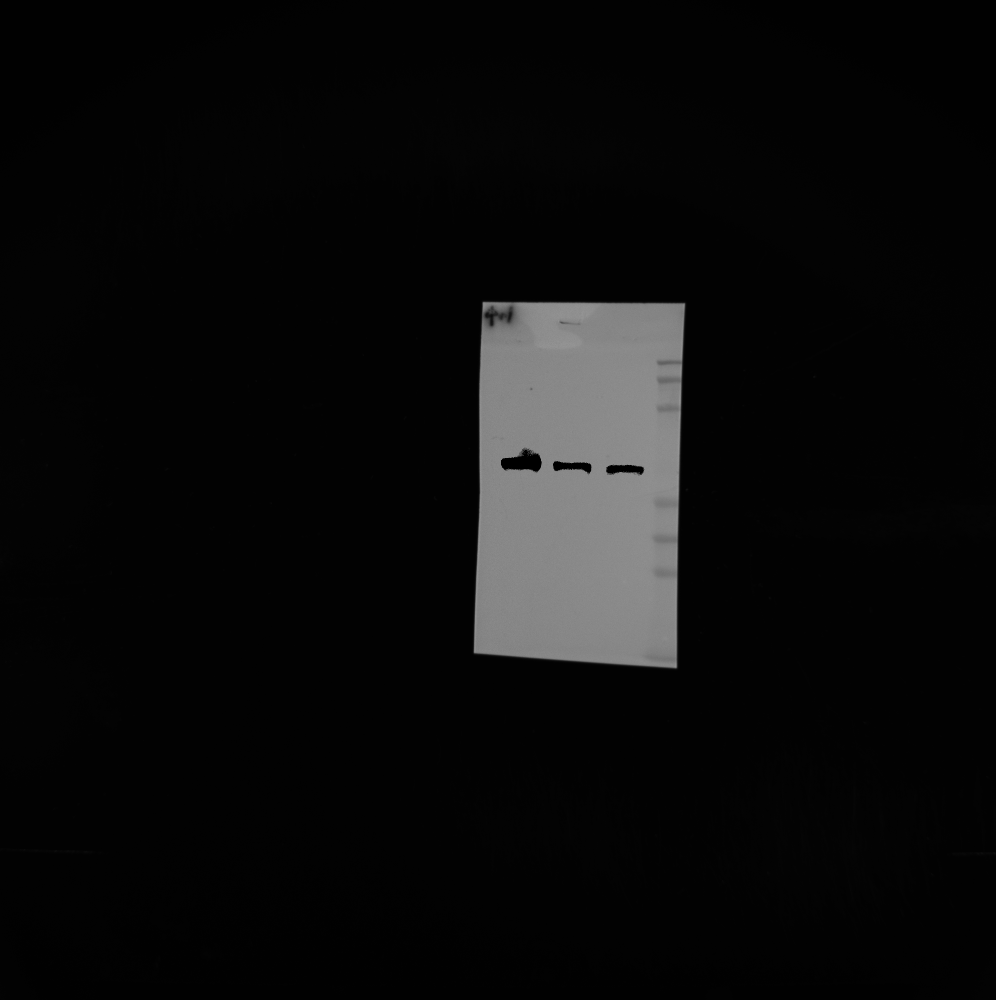


LAG-3 60kDa

**Extended Data Fig.2** The original blotting of LAG-3 in Fig.5E.


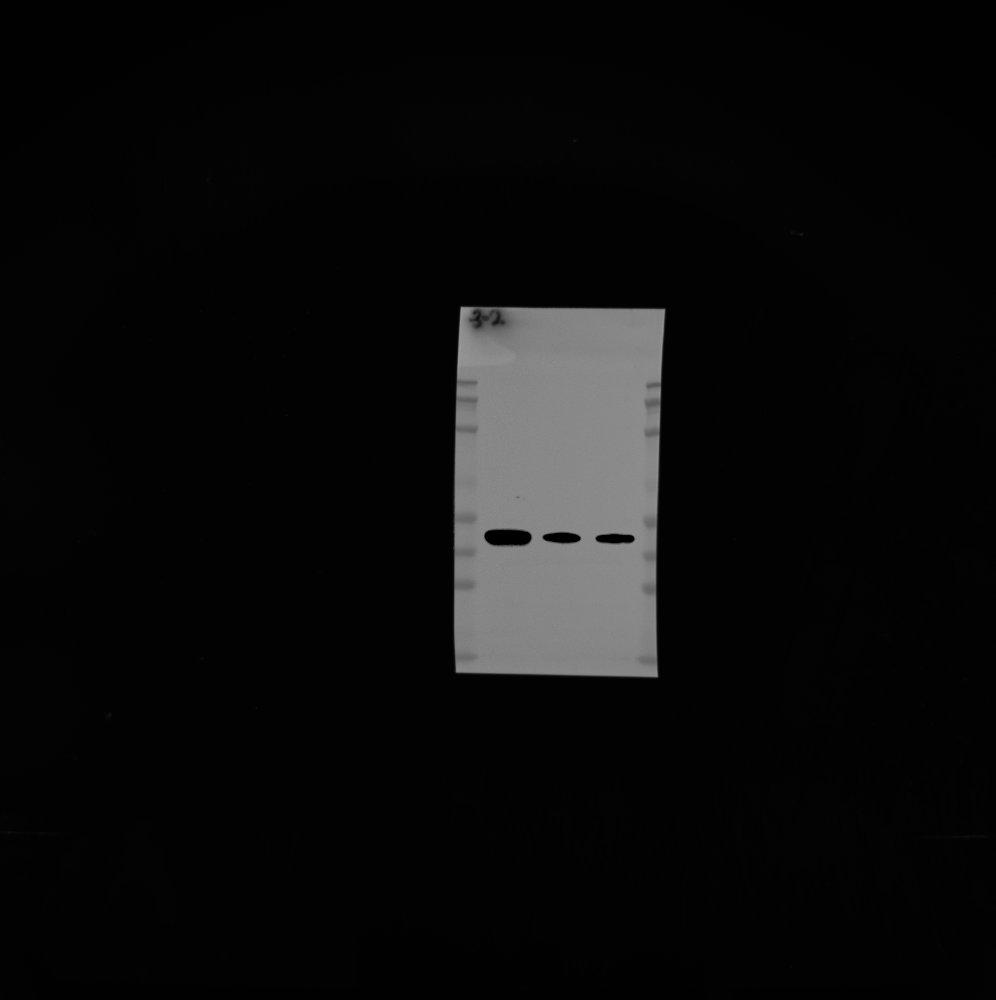


CD38 45kDa

CD38 45kDa

LAG-3 60kDa

**Extended Data Fig.3** The original blotting of CD38 in Fig.5E.


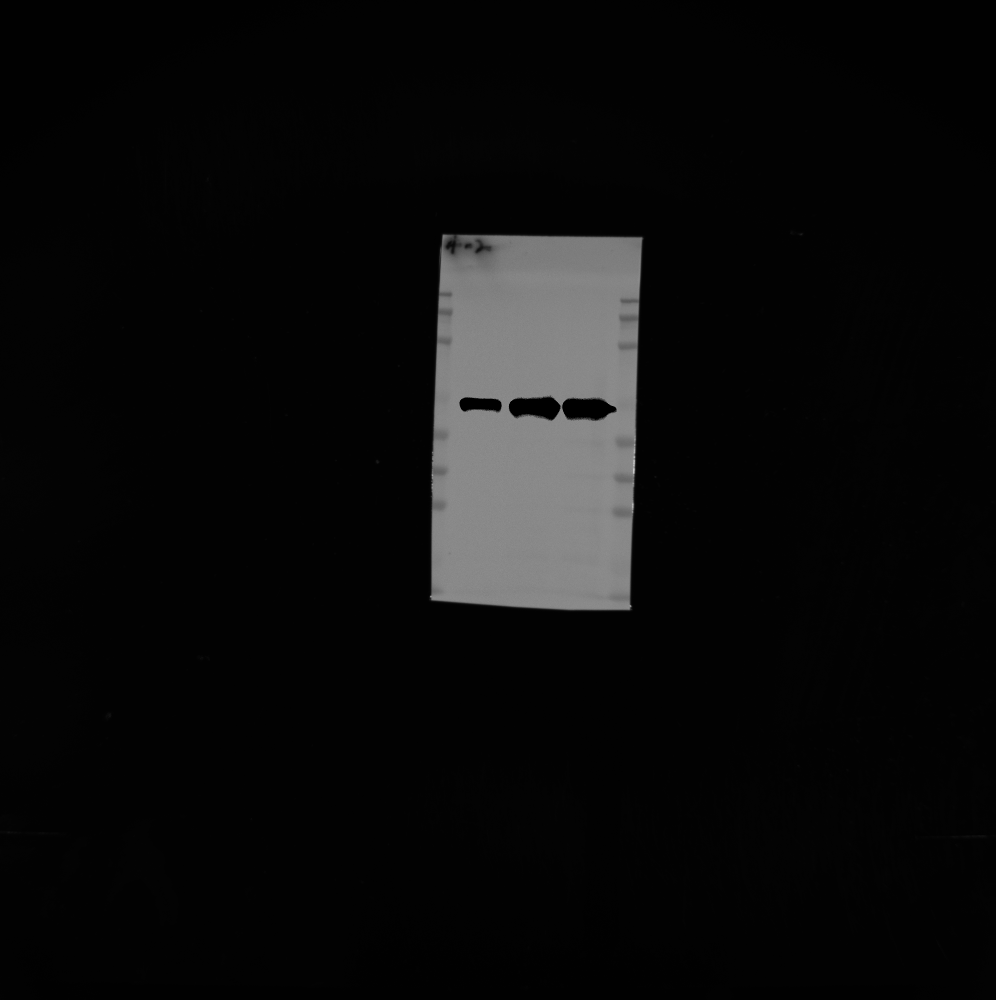


TCF-7 50kDa

**Extended Data Fig.4** The original blotting of TCF7 in Fig.5E.


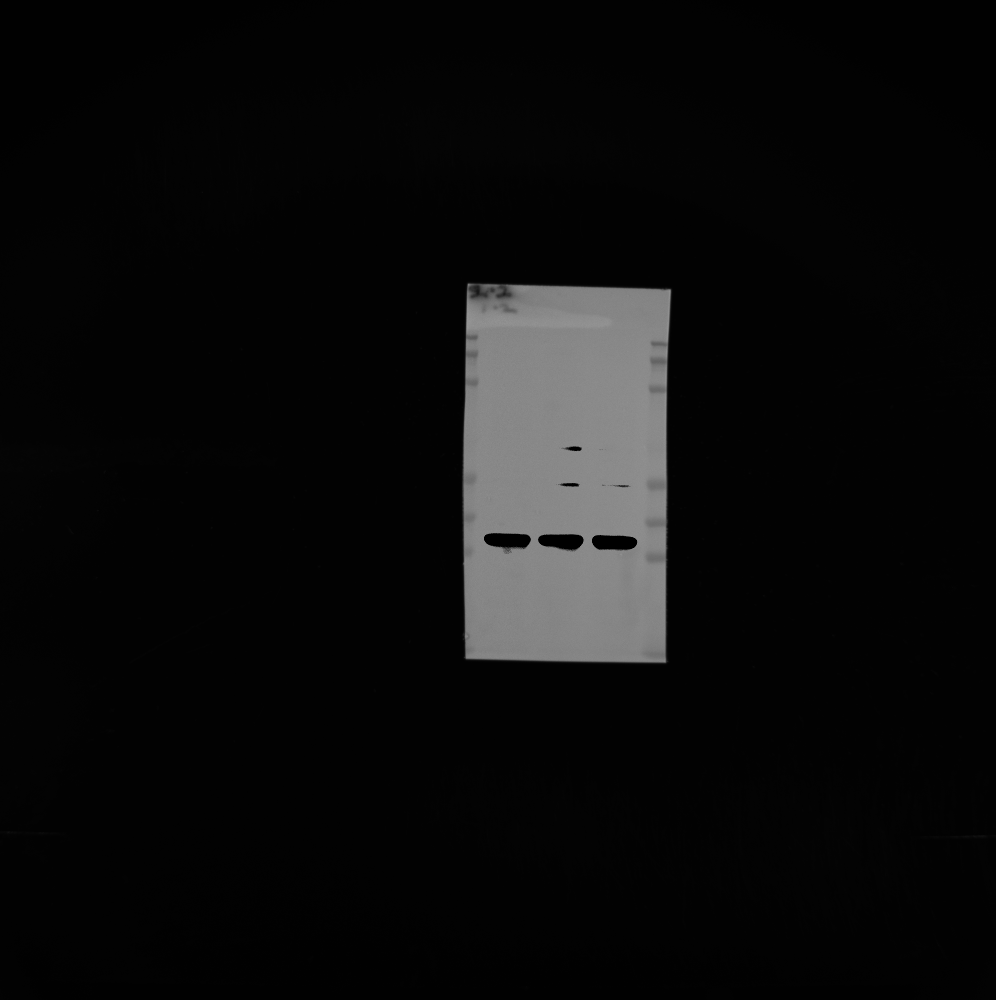


GAPDH 36kDa

**Extended Data Fig.5** The original blotting of GAPDH in Fig.5E.


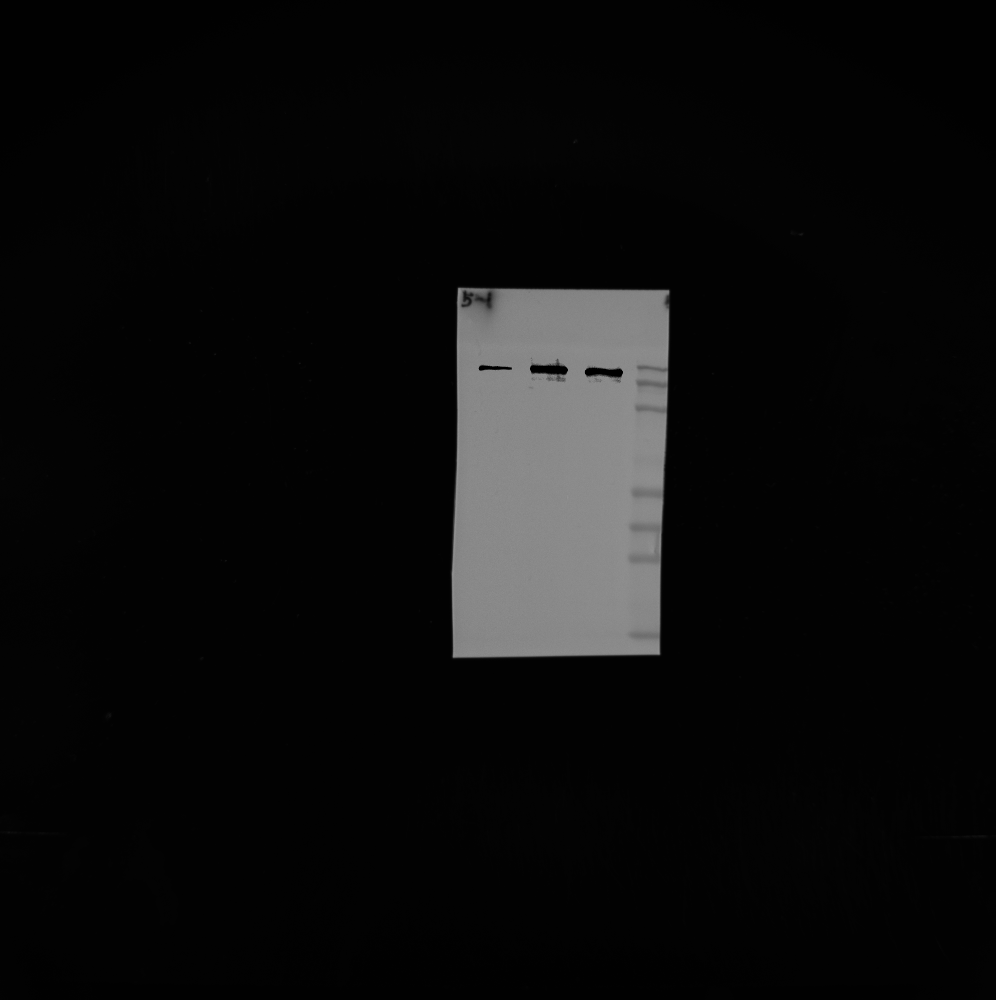


CD206 166kDa

**Extended Data Fig.6** The original blotting of CD206 in Fig.5F.


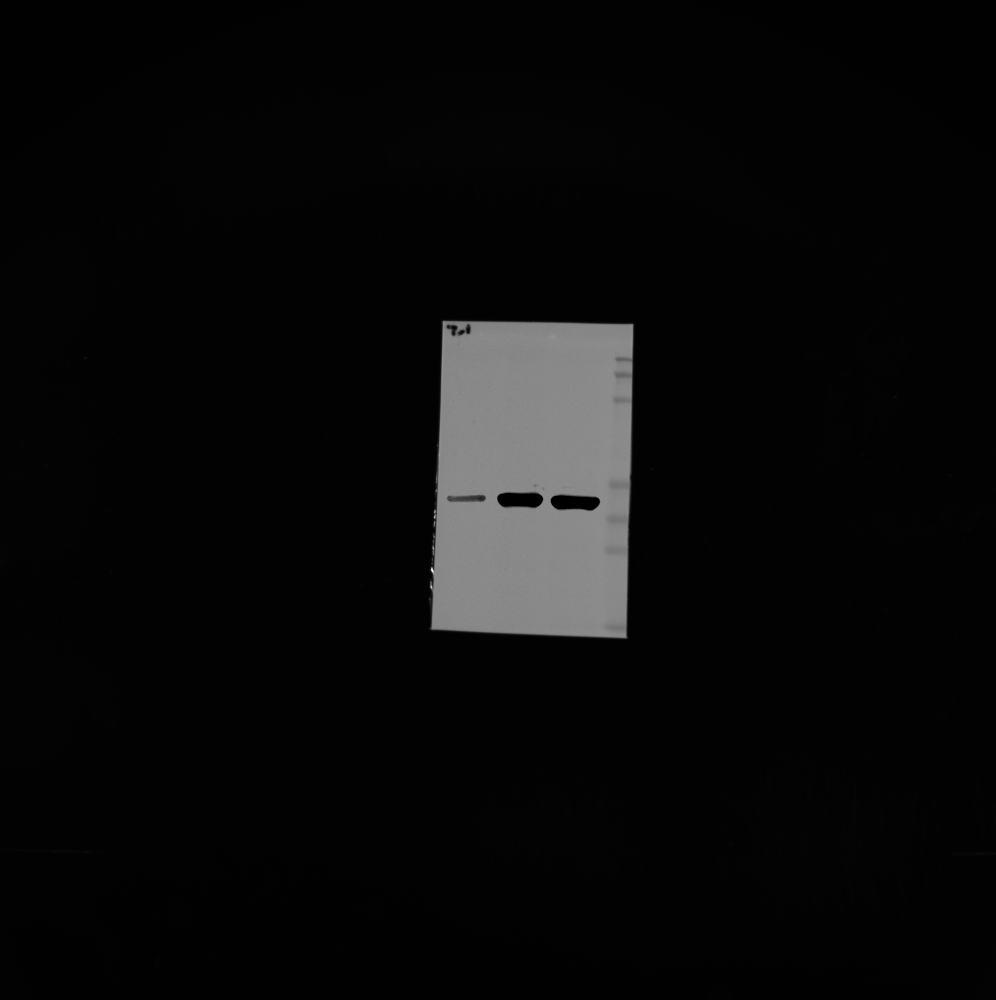


ARG-1 43kDa

**Extended Data Fig.7** The original blotting of ARG-1 in Fig.5F.


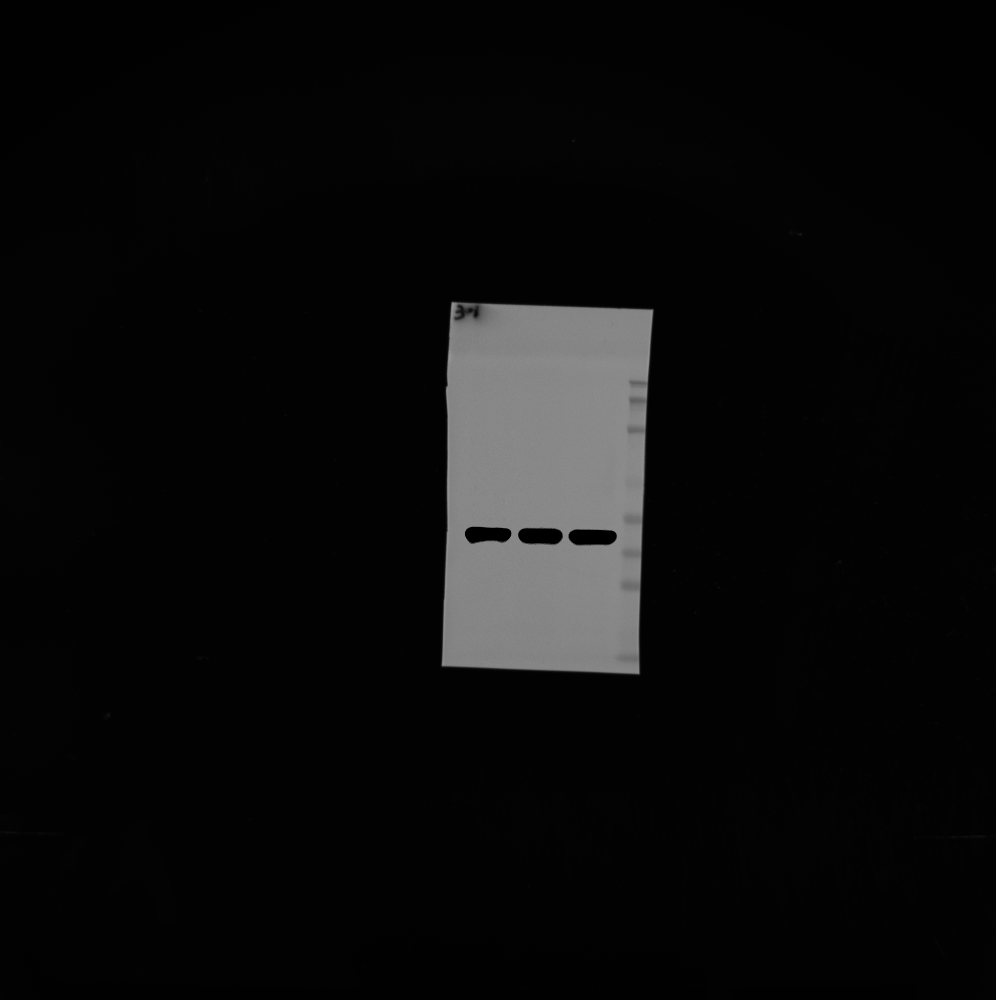


β-actin 42kDa

**Extended Data Fig.8** The original blotting of β-actin in Fig.5F.


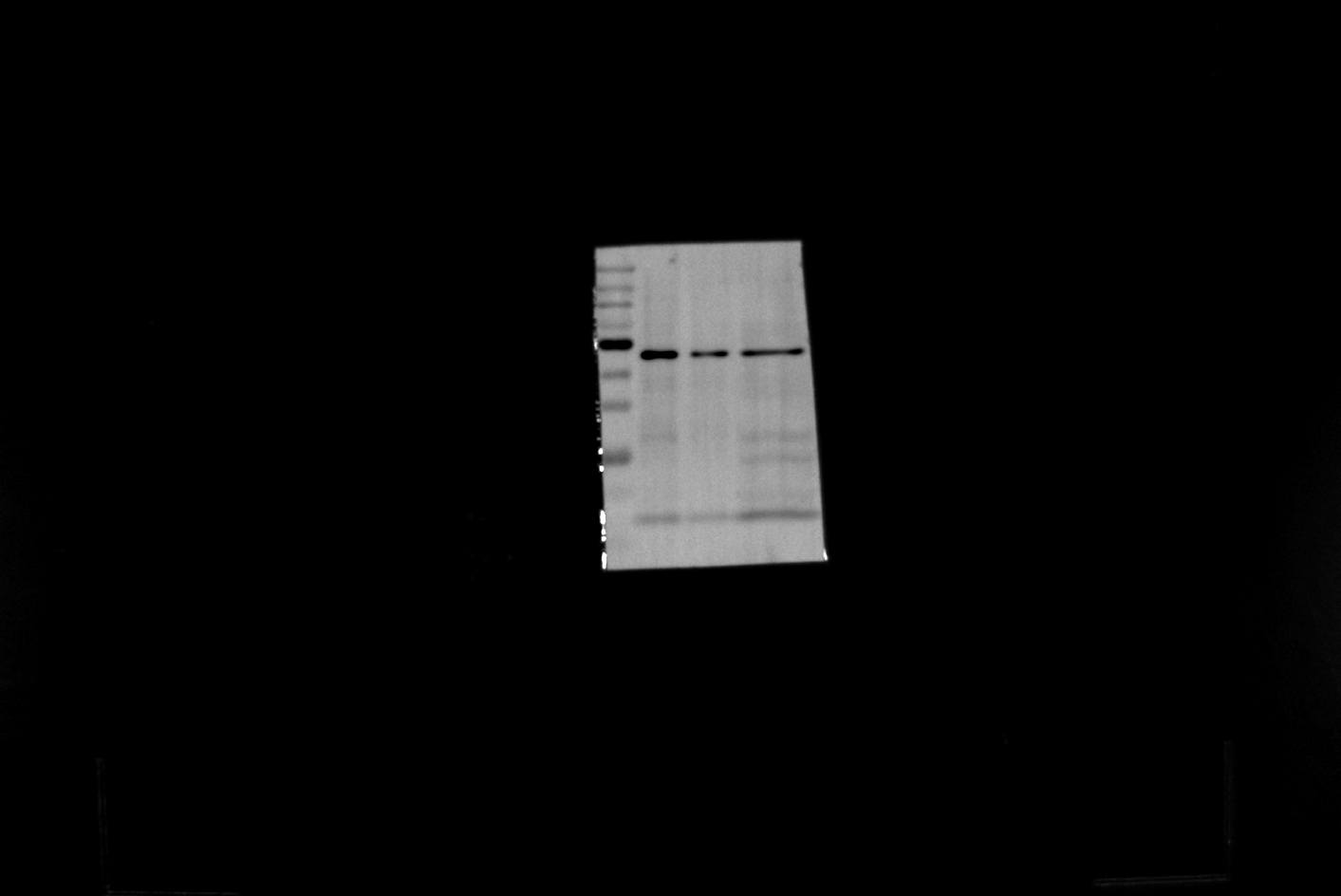


AKT 60kDa

**Extended Data Fig.9** The original blotting of AKT in Fig.7A.


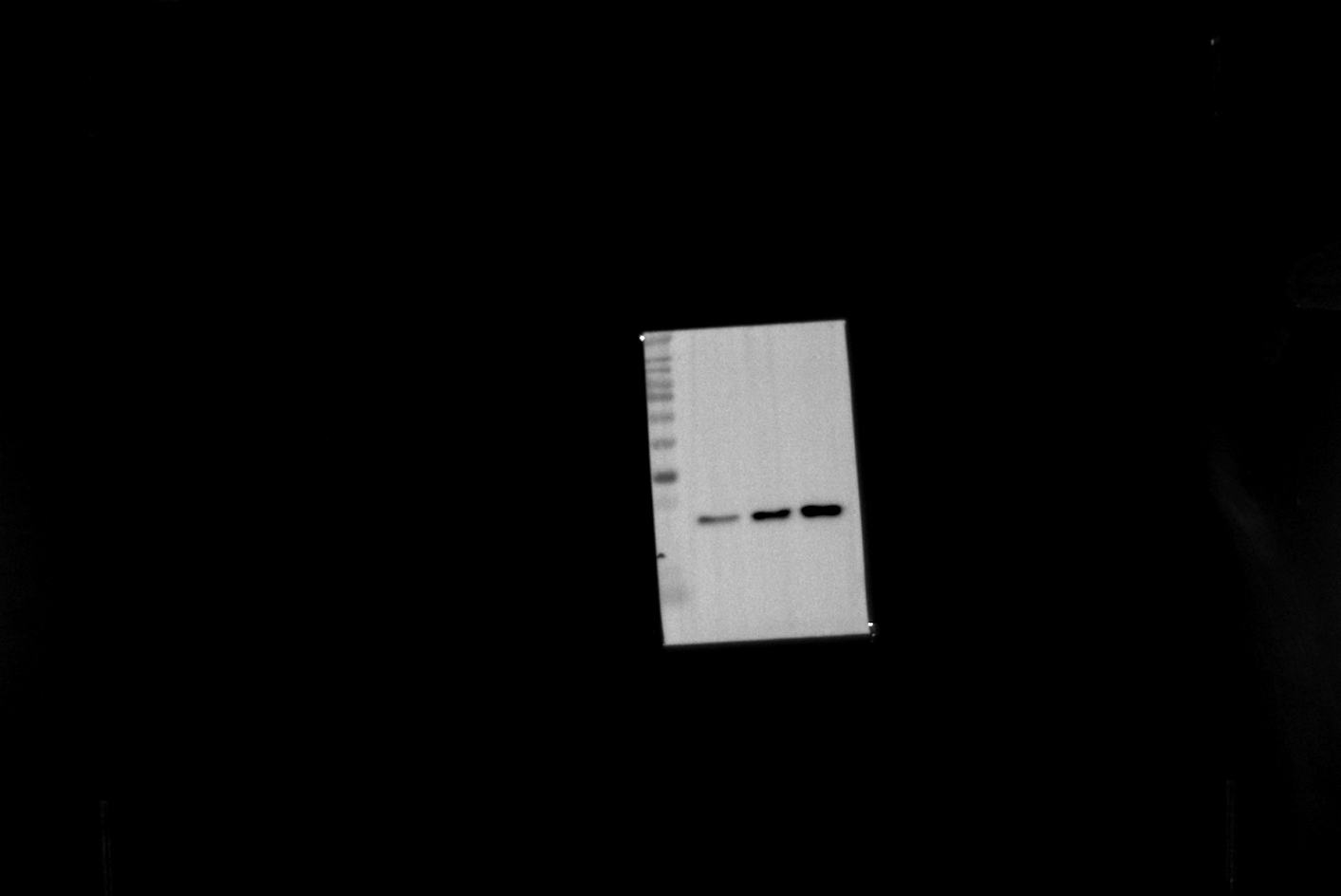


LC3-2 14kDa

**Extended Data Fig.10** The original blotting of LC3-2 in Fig.7A.


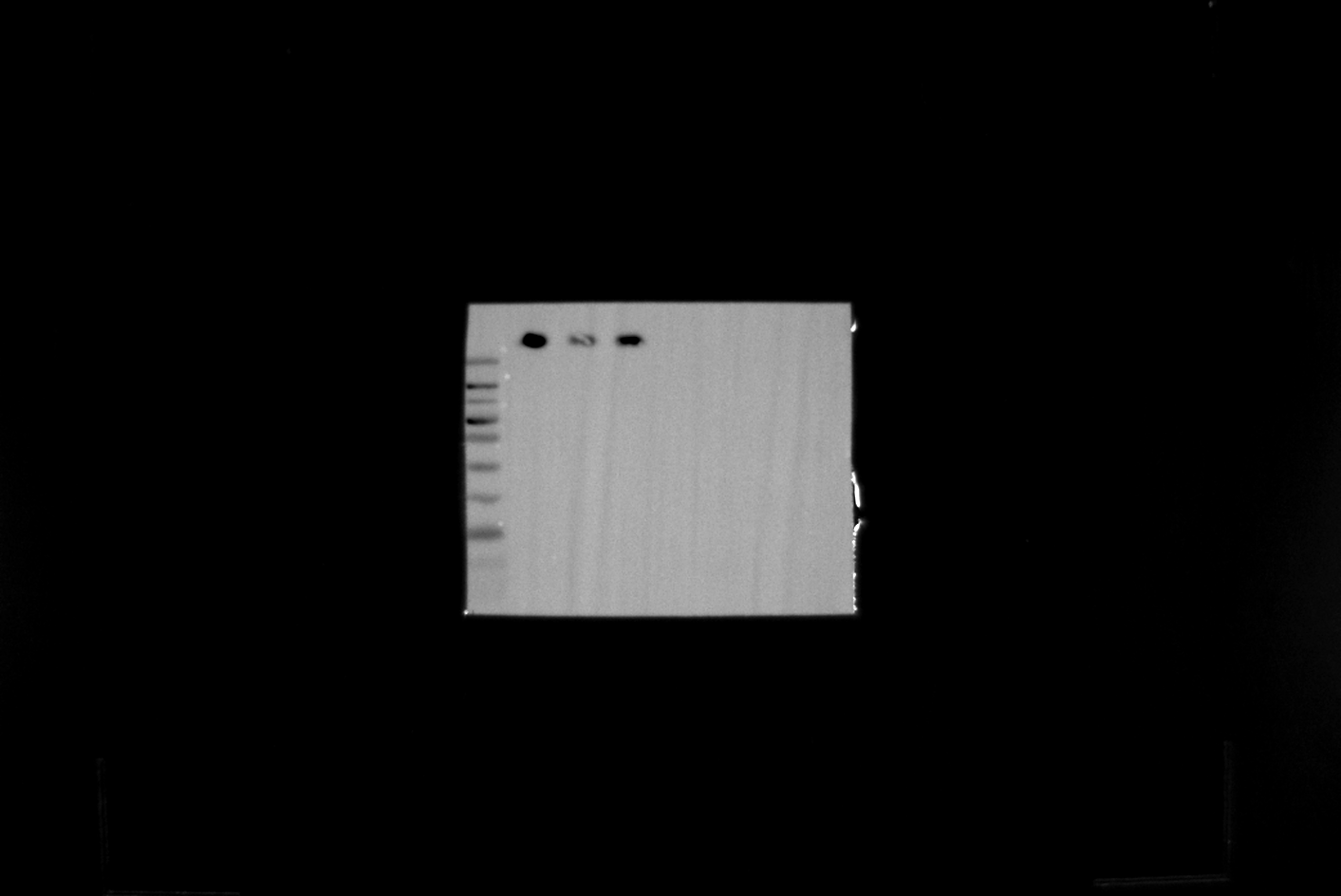


mTOR 289kDa

**Extended Data Fig.11** The original blotting of mTOR in Fig.7A.


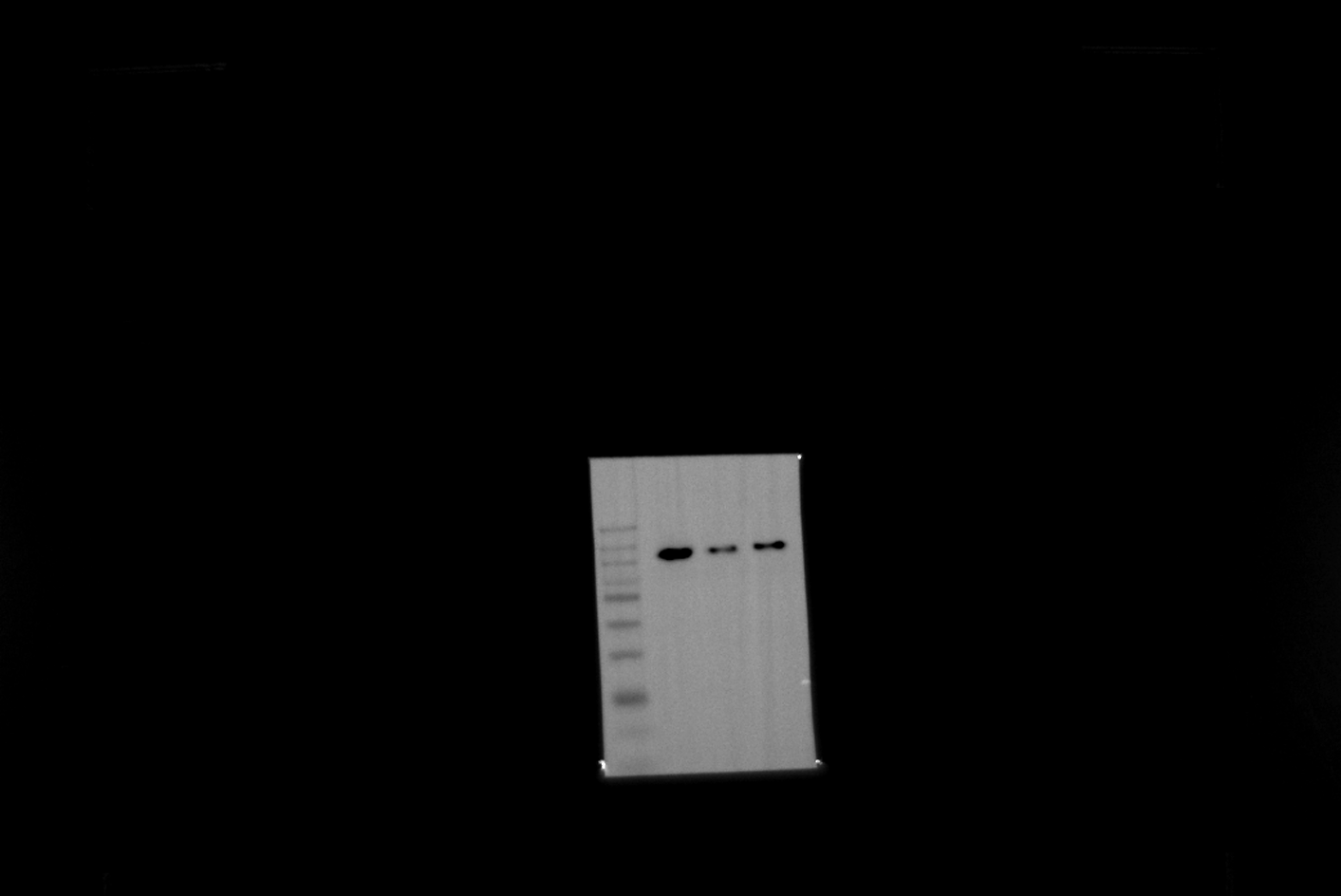


PI3K 110kDa

**Extended Data Fig.12** The original blotting of PI3K in Fig.7A.


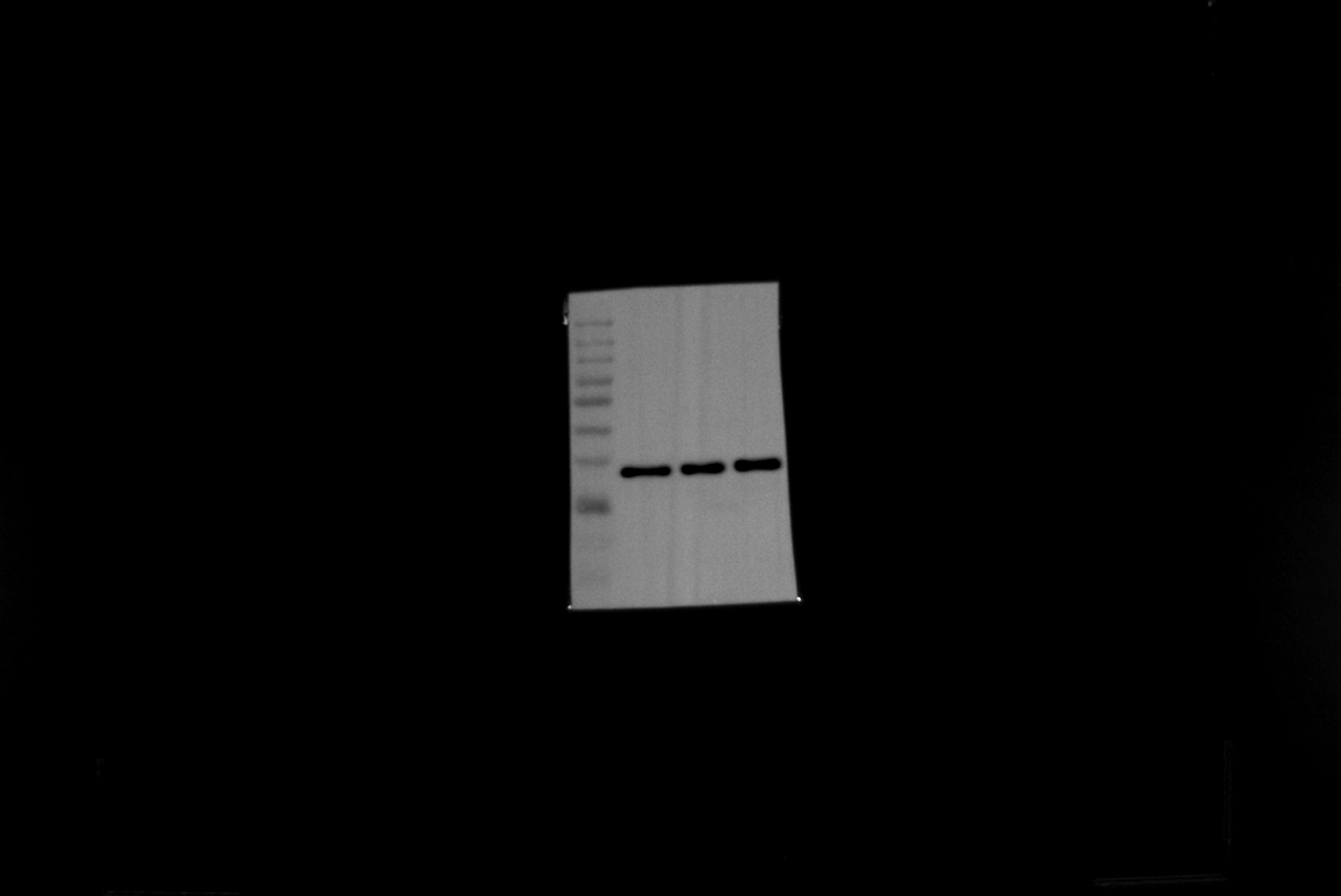


GAPDH 36kDa

**Extended Data Fig.12** The original blotting of GAPDH in Fig.7A.


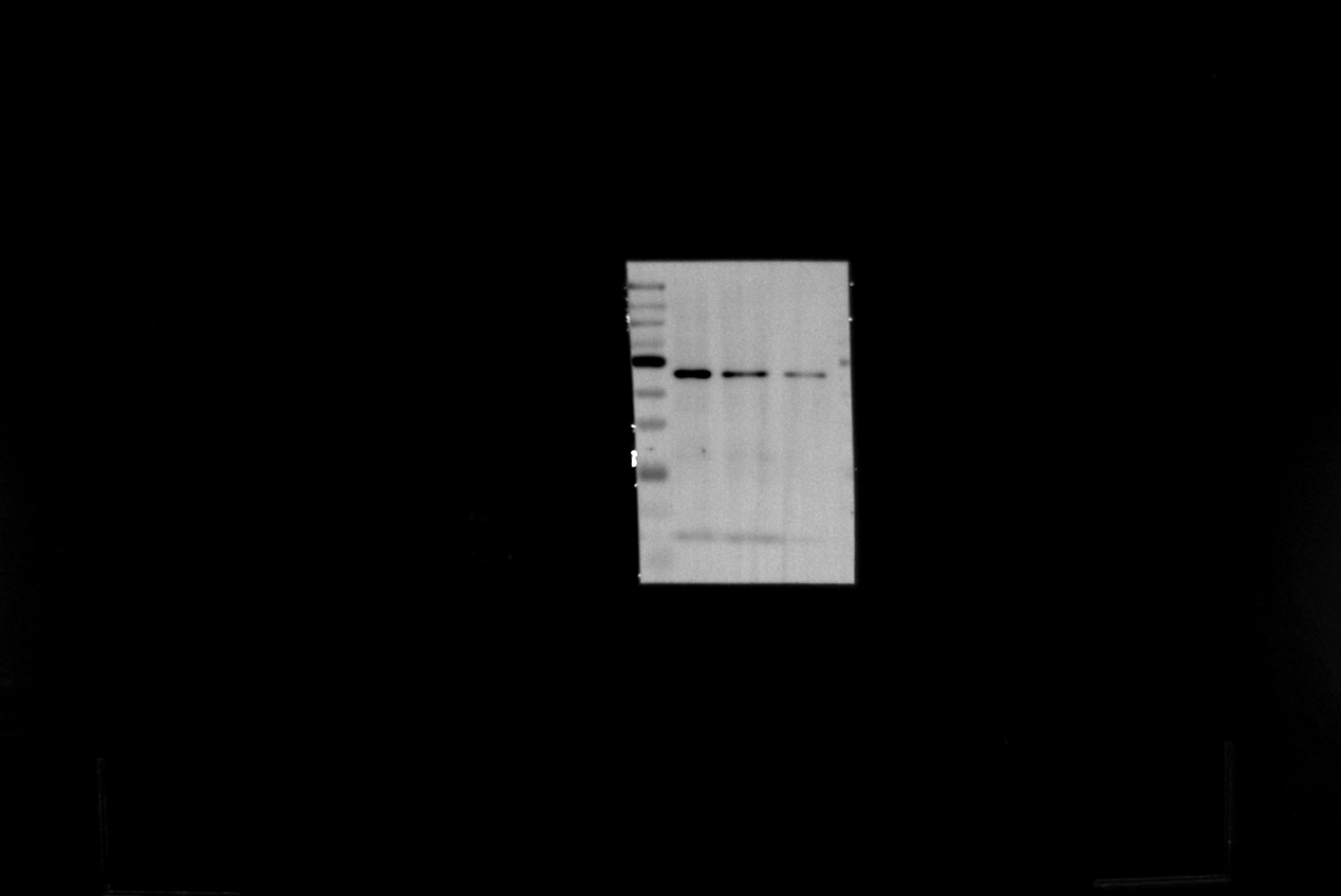


AKT 60kDa

**Extended Data Fig.13** The original blotting of AKT in Fig.7B.


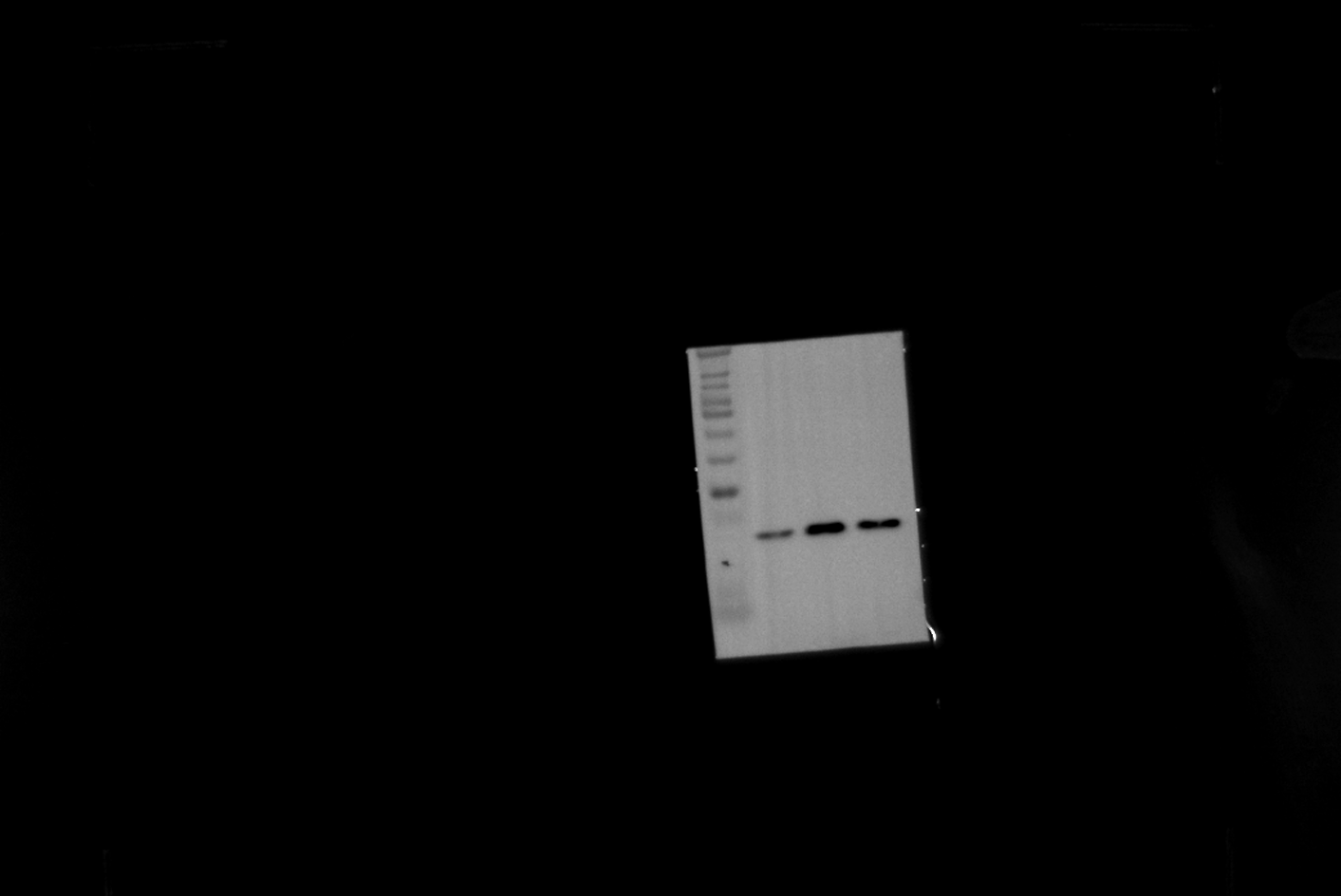


LC3-2 14kDa

**Extended Data Fig.14** The original blotting of LC3-2 in Fig.7B.


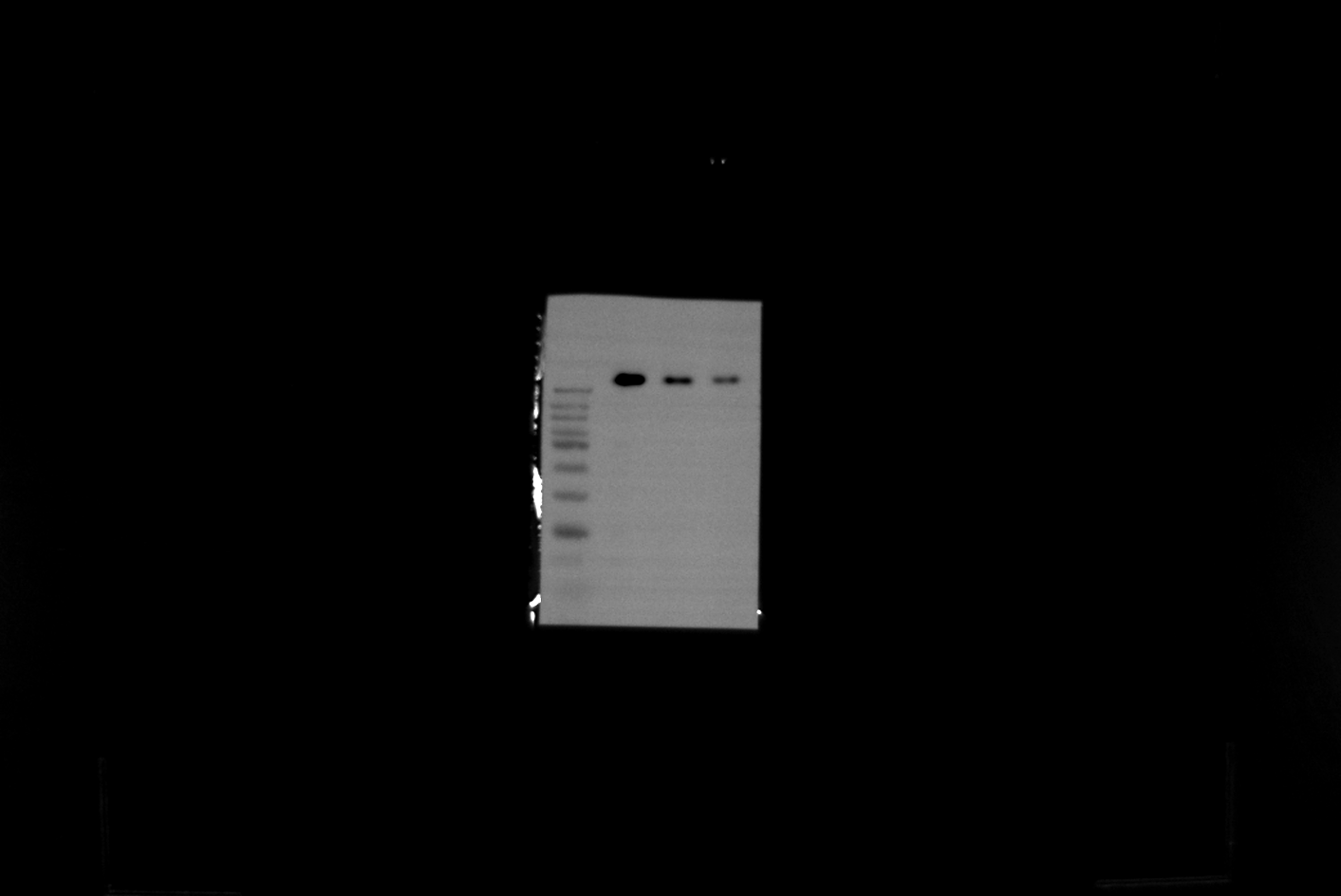


mTOR 289kDa

**Extended Data Fig.15** The original blotting of mTOR in Fig.7B.


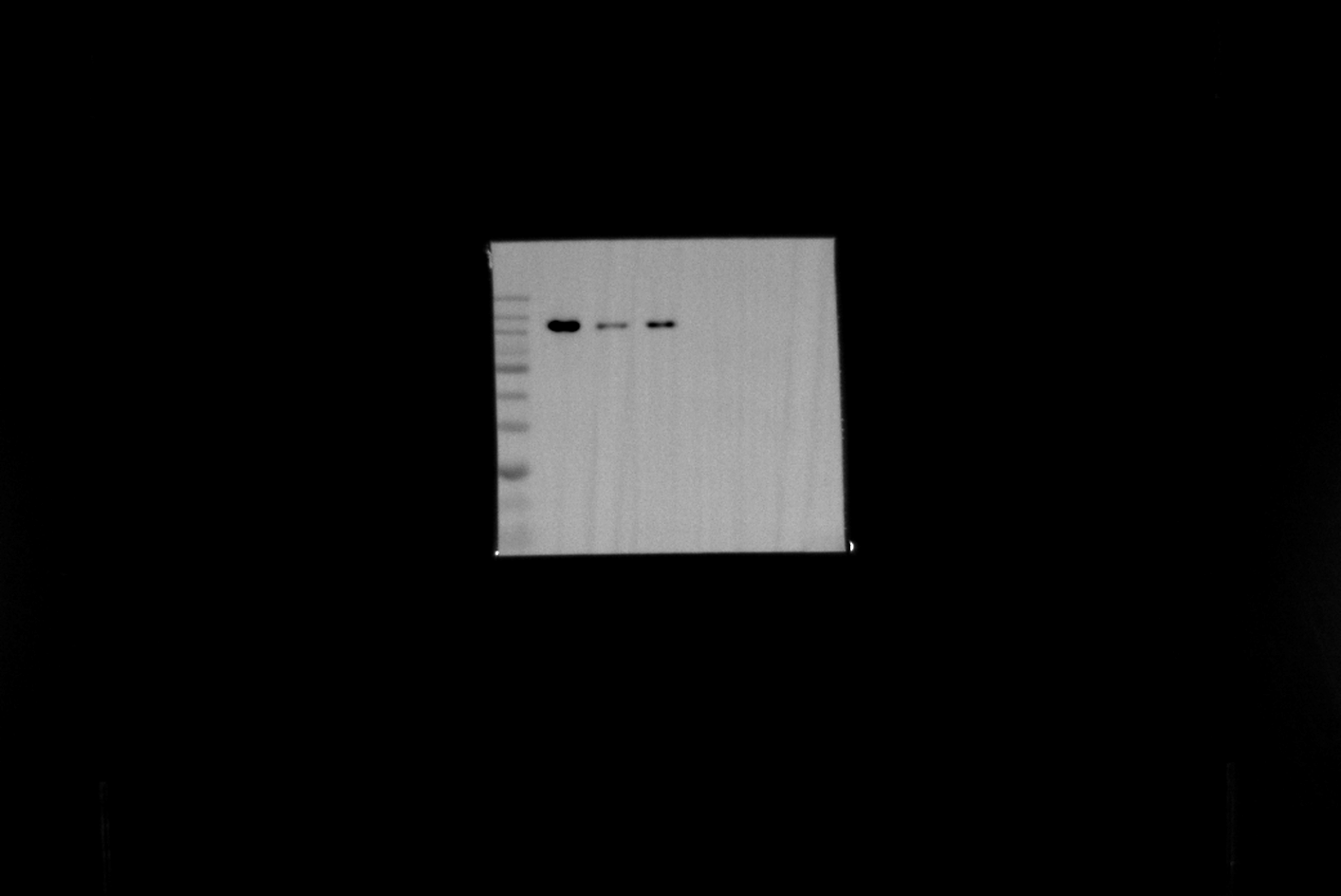


PI3K 110kDa

**Extended Data Fig.16** The original blotting of PI3K in Fig.7B.


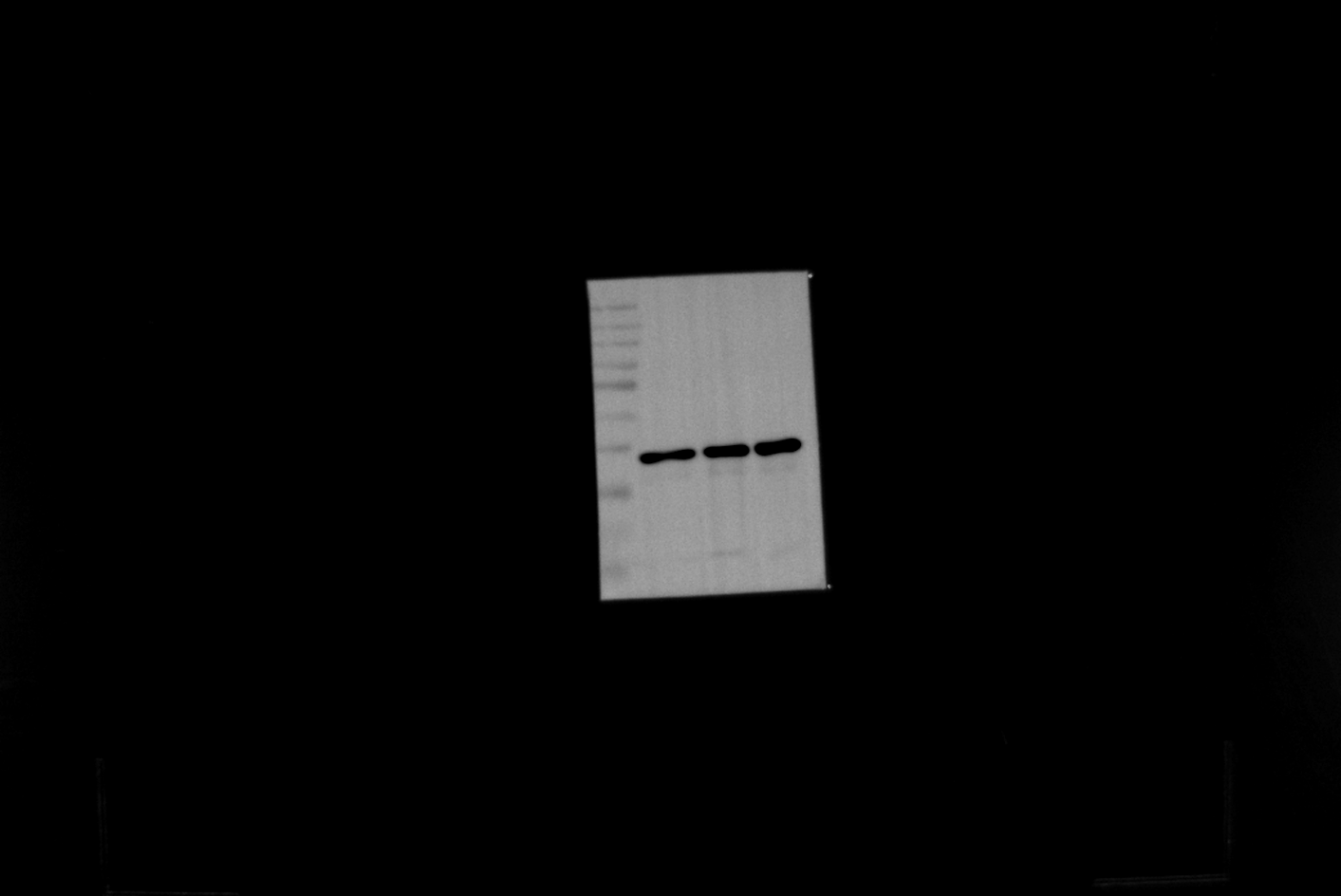


GAPDH 36kDa

**Extended Data Fig.17** The original blotting of GAPDH in Fig.7B.
